# Pretraining-improved Spatiotemporal graph network for the generalization performance enhancement of traffic forecasting

**DOI:** 10.1038/s41598-025-11375-2

**Published:** 2025-07-29

**Authors:** Xiangyue Zhang, Chao Li, Ling Ji, Yuyun Kang, Mingming Pan, Zhuo Liu, Qiang Qi

**Affiliations:** 1 School of Information Science and Engineering, Linyi University, Linyi, 276000 China; 2 Daopuyun (Shandong) Intelligent Technology Co., Ltd, Jinan, 265200 China; 3 School of Logistics, Linyi University, Linyi, 276000 China; 4 Linyi Research Institute of Trade Logistics Science and Technology Industry, Linyi, 276000 China

**Keywords:** Traffic forecasting, Pre-training, Spatio-temporal graph network, DDPM, State of space model, Computational science, Computer science

## Abstract

Traffic forecasting is considered a cornerstone of smart city development. A key challenge is capturing the long-term spatiotemporal dependencies of traffic data while improving the model’s generalization ability. To address these issues, various sophisticated modules are embedded into different models. However, this approach increases the computational cost of the model. Additionally, adding or replacing datasets in a trained model requires retraining, which decreases prediction accuracy and increases time cost. To address the challenges faced by existing models in handling long-term spatiotemporal dependencies and high computational costs, this study proposes an enhanced pre-training method called the Improved Spatiotemporal Diffusion Graph (ImPreSTDG). While existing traffic prediction models, particularly those based on Graph Convolutional Networks (GCNs) and deep learning, are effective at capturing short-term spatiotemporal dependencies, they often experience accuracy degradation and increased computational demands when dealing with long-term dependencies. To overcome these limitations, we introduce a Denoised Diffusion Probability Model (DDPM) as part of the pre-training process, which enhances the model’s ability to learn from long-term spatiotemporal data while significantly reducing computational costs. During the pre-training phase, ImPreSTDG employs a data masking and recovery strategy, with DDPM facilitating the reconstruction of masked data segments, thereby enabling the model to capture long-term dependencies in the traffic data. Additionally, we propose the Mamba module, which leverages the Selective State Space Model (SSM) to effectively capture long-term multivariate spatiotemporal correlations. This module enables more efficient processing of long sequences, extracting essential patterns while minimizing computational resource consumption. By improving computational efficiency, the Mamba module addresses the challenge of modeling long-term dependencies without compromising accuracy in capturing extended spatiotemporal trends. In the fine-tuning phase, the decoder is replaced with a forecasting header, and the pre-trained parameters are frozen. The forecasting header includes a meta-learning fusion module and a spatiotemporal convolutional layer, which facilitates the integration of both long-term and short-term traffic data for accurate forecasting. The model is then trained and adapted to the specific forecasting task. Experiments conducted on three real-world traffic datasets demonstrate that the proposed pre-training method significantly enhances the model’s ability to handle long-term dependencies, missing data, and high computational costs, providing a more efficient solution for traffic prediction.

## Introduction

 Traffic forecasting is a cornerstone of smart city development^1,2^. As a core technology of smart cities, traffic forecasting is not only crucial for optimizing traffic flow and alleviating road congestion, but also plays a significant role in reducing environmental pollution, enhancing public safety, and improving urban resource allocation. Accurate traffic forecasting, which facilitates decision-makers in responding more flexibly to changes in road conditions and enhances urban traffic efficiency^3^, is crucial for smart city development.

Graph learning methods have been demonstrated to play a crucial role across various domains. Among these methods, the application of stacked capsule autoencoders (SCAE) was proposed by Hong et al. to encode the parts and poses of facial images^4^. These encoded parts and poses are subsequently utilized to train templates and reconstruct the original facial image within the decoder. In addition, the SCAE was enhanced by incorporating a locality loss function, through which the internal relationships among similar samples are exploited. To achieve this, graph regularization is employed. In this manner, geometry-aware representations with improved quality can be computed. A novel 3D human pose estimation (HPE) architecture, termed the High-Order Graph Convolution Transformer (HOGFormer), was introduced by Xie et al. HOGFormer is composed of three core components: the Chebyshev Graph Convolution (CGConv) module, the Graph-based Dynamic Adjacency Matrix Transformer (GDAMFormer) module, and the High-Order Graph Convolution (HOGConv) module^5^. These advancements in graph-based learning provide valuable references for research in traffic prediction. Machine learning and deep learning are among the most important tools for traffic forecasting^6^. Numerous studies have confirmed the feasibility and significance of machine learning and deep learning in traffic forecasting^7^. The advantage of using machine learning and deep learning for traffic forecasting lies in their ability to automatically learn nonlinear relationships from large^8^, complex historical data, capture spatiotemporal dependencies^9^, and improve accuracy through multimodal data fusion. These methods can process dynamically changing data in real time^10^, provide efficient long-term predictions, and enhance accuracy and computing efficiency through automatic feature extraction and optimization algorithms. Traffic networks are typically represented as graph structures, where nodes correspond to traffic data points and edges represent correlations. Graph convolutional neural networks are used to extract spatiotemporal features and are often integrate with other modules, such as attention mechanisms^11,12^. Although existing GCNs and deep learning models have made notable progress in capturing short-term spatiotemporal dependencies, they exhibit significant limitations when addressing long-term dependencies, such as changes in traffic patterns over hours or even days. Capturing long-term spatiotemporal dependencies is crucial for the accuracy of traffic forecasting. However, existing models have limited capacity in this area, leading to a decrease in prediction accuracy, particularly when traffic patterns undergo substantial changes. In addition, current models typically require the processing of large-scale historical datasets and real-time traffic information. This not only increases computational costs but also complicates the model training and inference processes, thereby impacting both the efficiency and scalability of the models in practical applications.

**Fig. 1:**
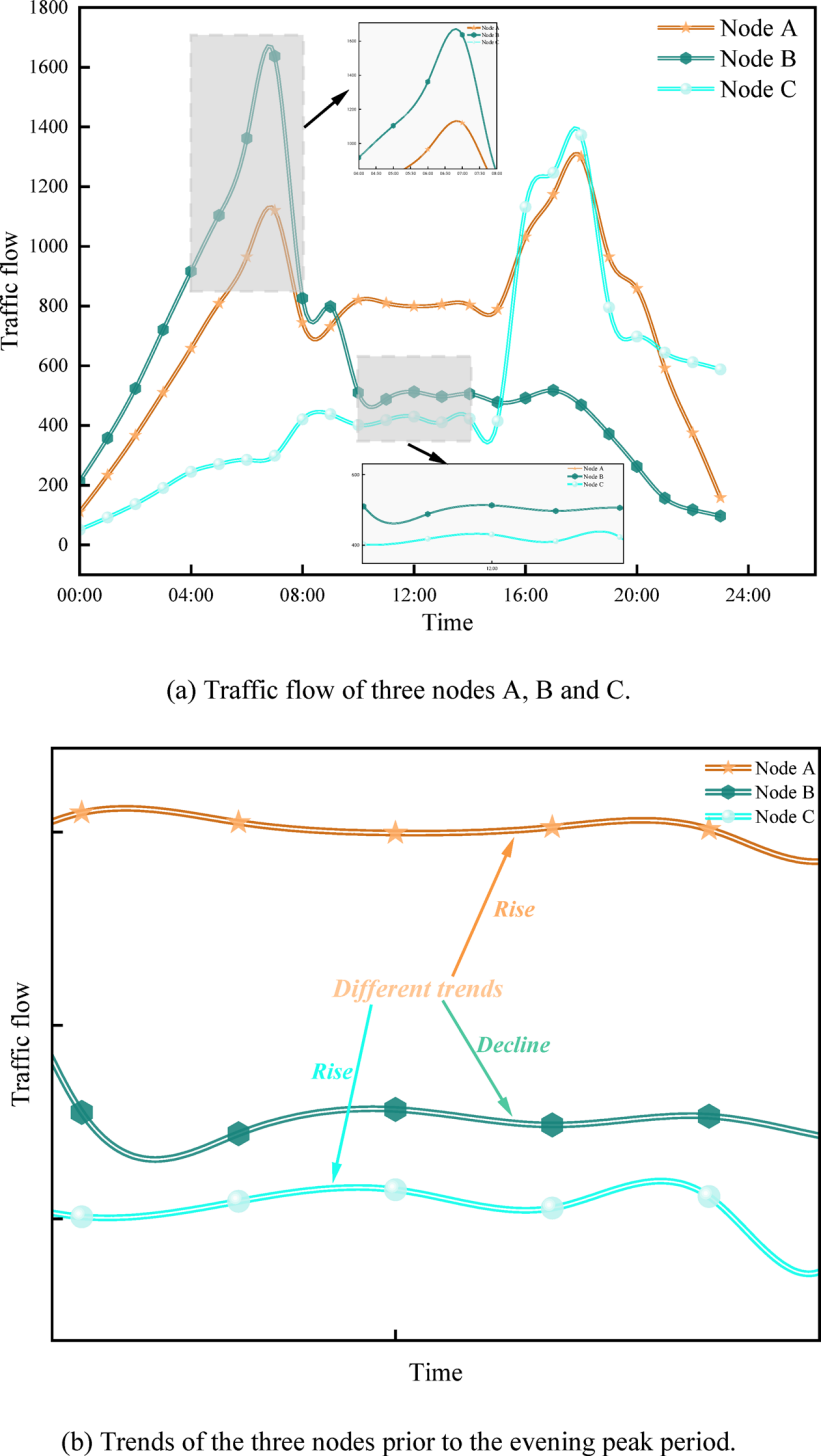
Traffic flow at three nodes within Linyi’s transportation network.

The disadvantage of using graph convolutional network methods for traffic forecasting is that it is only good at capturing short-term spatiotemporal data (the past one hour), but it is still far from enough to capture long-term spatiotemporal data (the past day or two days or even longer). This will lead to a decrease in the accuracy of the model’s traffic forecasting and misjudgment, as shown in Fig. 1. As shown in Fig. 1 (a), nodes A and C exhibit highly similar trends around 07:00. During the subsequent evening peak period, node A peaks again, while node B shows a stable or declining trend. However, while nodes A and C exhibit similar trends during the evening peak, the trend of node C prior to this period differs significantly from that of node A. As shown in Fig. 1 (b), analyzing only the 6 h before the evening peak may lead the model to incorrectly predict a traffic peak at node B due to the high similarity between nodes A and C. In a word, this analysis highlights the limitations of focusing solely on short-term traffic flow and underscores the need to consider long-term data perspectives.

While current models, which can effectively capture and analyze long-term spatiotemporal data^13–16^, also increase in computational cost linearly with the continuous embedding of data, this aspect should be considered. Training strategies in natural language processing (NLP)^17,18^, which have gained widespread attention due to their effectiveness in capturing relationships within long sequences of words and sentences, are fundamental to the field. The core principle of pre-training involves masking part of the sequence data, which compels the model to reconstruct the masked portion or predict subsequent data. Capturing relationships in long sequences for traffic forecasting is similar to natural language processing, enabling the use of similar pre-training strategies. This approach leverages a unified pre-trained model backbone to handle various downstream tasks. However, directly applying NLP pre-training methods to traffic forecasting is not feasible without modification. First, in natural language processing, each word carries distinct information, and sentences composed of multiple words convey clear semantics. In contrast, each traffic data point contains limited information. Second, as shown in Fig. 1(b), each traffic data point contains numerical noise, which hinders the model’s ability to capture contextual trends. Additionally, the extremely long input sequences impose a heavy computational burden, and the temporal and spatial dependencies in traffic data must be accounted for. On the other hand, by introducing the Mamba module, a selective mechanism (selective SSM) is employed to capture multivariate correlations^19^, thereby enhancing computational efficiency.

To address these challenges, this study proposes an enhanced pre-training method, the ImPreSTDG, designed to effectively extract long-term spatiotemporal features through data masking and recovery tasks. This approach aims to improve the accuracy and practicality of traffic prediction by reducing computational costs and enhancing the model’s robustness in scenarios with missing data. The structure of this study is shown as follows. In Section II, the related literature is reviewed. In Section III, methodology of this study is introduced. In Section IV, the details of the experiment details are described. Finally, in Section V, this study is summarized and future research directions are proposed.

## Related work

**Fig. 2:**
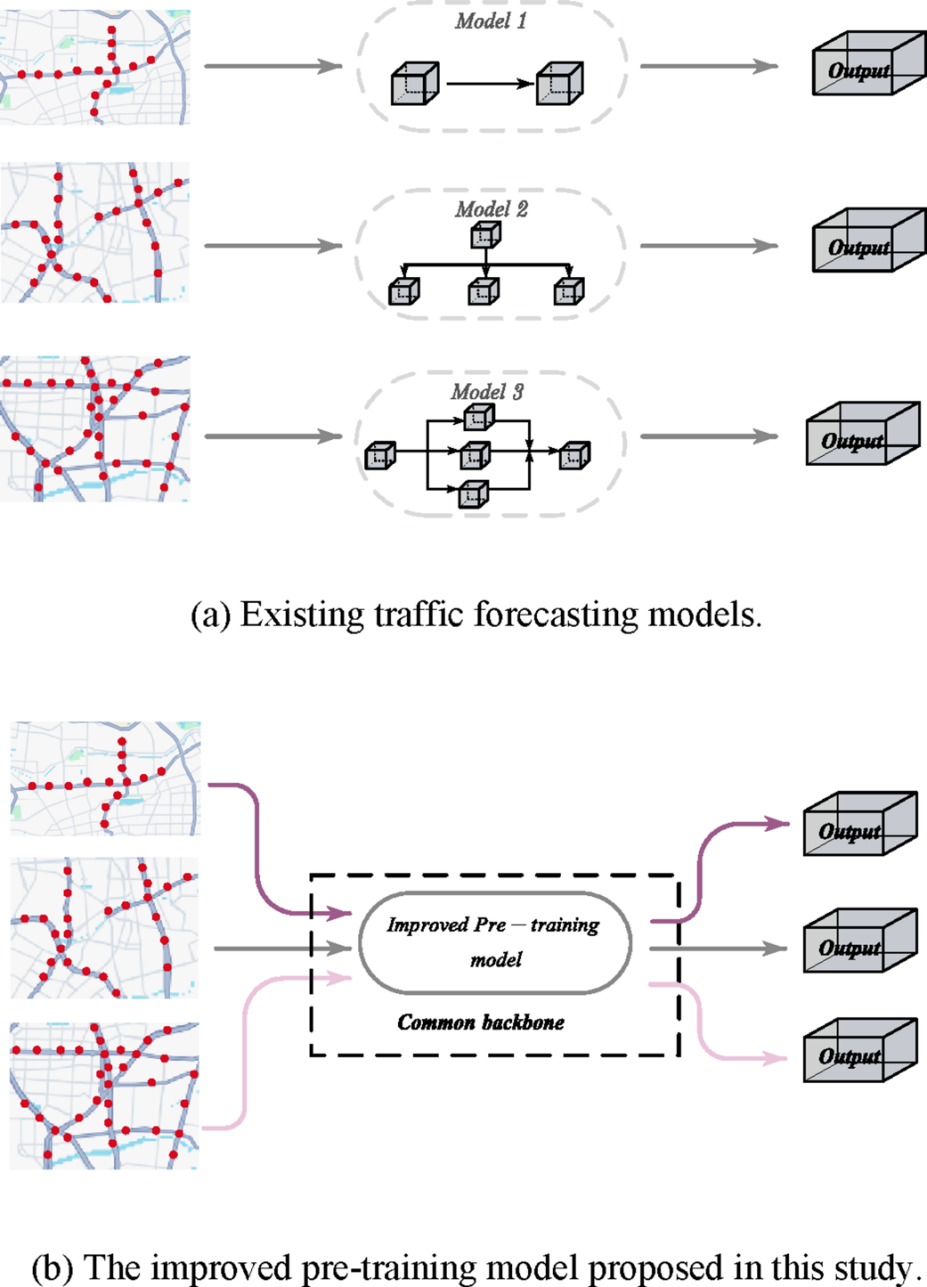
Comparison of existing traffic forecasting models with the improved pre-trained model proposed in this study.

Although many advanced traffic prediction models, such as those based on GCN and Spatiotemporal Graph Neural Networks (STGNN), have demonstrated promising results in specific scenarios, they often struggle to adapt to diverse traffic networks and varying environmental conditions. When applied to new cities or different types of traffic data, these models exhibit limited generalization capability. Furthermore, the prevalent issue of missing data in traffic forecasting—especially due to sensor failures, extreme weather, and similar factors—further complicates model performance. Existing models often rely on complete data inputs and lack effective mechanisms for handling missing data, significantly reducing their reliability in real-world applications. In summary, this study aims to enhance the model’s generalization ability and improve its resistance to data disturbances from various sources (as shown in Fig. 2).

### Traditional traffic forecasting models

Traditional traffic forecasting models form the foundational basis upon which modern predictive frameworks have been developed. These approaches, which range from statistical techniques to shallow machine learning methods and eventually to early deep learning paradigms, were initially successful in modeling short-term temporal patterns^20^. However, they have often been found inadequate when tasked with capturing the complex, high-dimensional, and nonlinear spatiotemporal dynamics that characterize real-world traffic systems.

One of the most fundamental methods that has been widely used is the K-Nearest Neighbors (KNN) algorithm, in which predictions are made based on the similarity between historical patterns. As was demonstrated by Zhang et al.^21^, KNN is capable of achieving acceptable performance under stable traffic conditions by identifying similar temporal segments from historical traffic flow data. However, because this method is entirely data-driven and lacks parametric interpretability, its effectiveness is significantly diminished when applied to volatile traffic patterns or scenarios with missing data.

In an effort to overcome these limitations, Gated Recurrent Units (GRUs) were introduced into traffic prediction tasks. A GRU-based model was proposed by Shu et al.^22^, in which nonlinear temporal dependencies are efficiently captured while computational overhead—typically associated with Long Short-Term Memory (LSTM) networks—is reduced. The model’s ability to selectively retain or discard historical information through its gating mechanisms made it particularly well-suited for short-term forecasting. Nonetheless, similar to KNN, GRU-based models do not incorporate spatial structure, as traffic time series are treated as independent sequences without consideration of the underlying connectivity among road segments. A subsequent evolution was realized with the application of Convolutional Neural Networks (CNNs), through which localized temporal features were extracted in a windowed fashion. CNNs were employed by Zhang et al.^23^ to detect temporal trends in traffic flow data, enabling the extraction of hierarchical features from large-scale datasets. However, since CNNs are inherently designed for Euclidean data structures, they are ill-suited to capture the irregular spatial topologies characteristic of road networks. Furthermore, due to their fixed receptive fields, their ability to model long-range temporal dependencies is inherently constrained.

In response to these challenges, graph-based learning approaches were explored, as they are better aligned with the non-Euclidean nature of traffic systems. A STGNN was proposed by Wang et al.^23^, in which spatial and temporal dependencies are jointly captured through the integration of graph convolutional operators with sequence modeling techniques. In their approach, nodes are defined as sensor locations and edges as roadway connections, allowing for a more dynamic representation of traffic networks. Despite these advantages, such architectures have been observed to suffer from accuracy degradation when long input sequences are used or when data is missing, largely due to their reliance on static graph structures and limited sequence modeling capacity.

To better accommodate temporal heterogeneity and periodic traffic behavior, a Spatio-temporal Sequence-to-Sequence Network (STSSN) was introduced by Cao et al.^24^. This model, which was built on an encoder-decoder architecture, incorporates Enhanced Diffuse Convolutional Networks (EDCNs) and Temporal Convolutional Networks (TCNs) to model both daily and weekly traffic periodicities. An encoder-decoder attention (EDA) mechanism was also integrated to enable long-range temporal modeling. However, due to its architectural complexity and dependence on clean input data, the model becomes vulnerable when applied in real-world scenarios involving sensor failures or weather-induced data interruptions.

To specifically address the widespread issue of missing data, DI-LSTM was proposed by Li et al.^25^, in which LSTM networks are combined with a temporal damping mechanism to interpolate missing values prior to forecasting. Although this model exhibits improved robustness in handling data discontinuities, its decoupling of imputation and prediction introduces the risk of error propagation between stages.

More recent efforts have attempted to integrate multiple components to better capture spatial structure and temporal dynamics. For instance, the Task Efficient Graph Attention Network (TE-GAT) has been used to enhance Di-GraphGAN for graph learning, while Temporal Context Attention (TCA) mechanisms have been introduced to dynamically reweight historical data^26^. Although such composite models offer enhanced adaptability, they often impose substantial computational demands and require carefully calibrated architectures to generalize across diverse urban networks.

In summary, while a wide range of models—from KNN to DI-LSTM—have laid the groundwork for data-driven traffic forecasting, they remain limited in their capacity to model long-range dependencies, to process non-Euclidean spatial inputs, and to function robustly in the presence of incomplete or noisy data. These limitations underscore the necessity of developing unified, pretrainable architectures that can integrate spatial reasoning, deep temporal modeling, and adaptability to real-world conditions—capabilities that are addressed by the framework proposed in this study.

### Graph-based and Attention-enhanced models

To address the limitations inherent in traditional traffic forecasting methods—particularly in modeling spatial dependencies and in accommodating the non-Euclidean nature of road networks—researchers have increasingly adopted graph-based learning paradigms. In these approaches, the topological structure of traffic systems is explicitly leveraged by representing roads, intersections, or sensors as nodes, and by modeling their interconnections as edges within a graph. Among such methods, Graph Convolutional Networks (GCNs) have emerged as a foundational technique through which spatial correlations can be effectively learned from graph-structured data.

One of the early and influential developments in this direction was the introduction of STGNNs, in which GCNs are extended through integration with temporal modeling modules. For example, a model was proposed by Wang et al.^23^, in which spatial graph convolutions are combined with sequential learning units to simultaneously capture intra-temporal and inter-node dependencies within a unified framework. By moving beyond the assumptions of Euclidean space, these models are capable of learning richer representations of urban traffic dynamics. Nevertheless, several limitations remain. Many of these models rely on static or manually constructed adjacency matrices, are sensitive to the quality of the underlying graph structure, and struggle with scalability when faced with long temporal sequences or incomplete and noisy data inputs.

In response to the need for improved temporal modeling, sequence-to-sequence architectures augmented with attention mechanisms have been explored. A representative example is the STSSN)introduced by Cao et al.^24^, which incorporates EDCNs for spatial processing and TCNs for extracting temporal features. A central innovation of this model lies in its EDA mechanism, through which long-range temporal dependencies are captured while mitigating the risk of error accumulation. However, despite the sophistication of its design, STSSN remains highly sensitive to input data quality and often requires carefully customized components to adapt to varying traffic scenarios.

To address the challenge of missing or corrupted sensor data, the DI-LSTM model was proposed by Li et al.^25^, wherein a temporal damping mechanism is integrated within a recurrent framework to estimate missing values and stabilize time-series reconstructions. While this model enhances robustness to incomplete data, it treats interpolation and forecasting as two disjoint tasks, thereby risking the propagation of errors across stages and lacking a globally optimized mechanism for joint modeling.

Subsequent advancements have led to the adoption of graph attention mechanisms, which allow for adaptive, data-driven weighting of neighboring nodes. For instance, the Task-Efficient Graph Attention Network (TE-GAT), developed in conjunction with Di-GraphGAN^26^, incorporates attention layers to flexibly model traffic dynamics based on node-level interactions. When combined with the TCA module, this architecture is capable of capturing both spatial irregularity and temporal evolution in a context-aware manner. Nonetheless, the deployment of such models often results in high computational overhead due to the multiple attention layers involved, and their performance tends to be highly dependent on careful tuning across datasets or urban contexts.

Although these models collectively represent meaningful progress in learning complex spatiotemporal patterns, they continue to face three core limitations:

1. Long-sequence temporal modeling remains constrained by architectural depth and computational cost, particularly in Transformer-based variants.
2. Handling noisy or incomplete data is frequently treated as a preprocessing step external to the core learning pipeline, often via separate imputation modules.
3. Generalization to unseen domains is weak, as many models are prone to overfitting to the structural properties of the training graph, thereby limiting their transferability.

These persistent challenges highlight the need for a unified architectural solution—one that can efficiently model long temporal sequences, inherently incorporate spatial structures, and robustly handle incomplete data, while also enabling pre-training and transfer across heterogeneous datasets. This necessity forms the foundation for the introduction of state-space modeling techniques and diffusion-based generative frameworks, both of which provide the theoretical and practical underpinnings for the pretraining strategy proposed in this study.

### Pretraining and Long-sequence modeling paradigms

The increasingly complex structure of urban traffic systems has introduced significant challenges for traditional forecasting models, particularly when long-term dependencies, large-scale spatiotemporal data, and heterogeneous input conditions must be addressed. Most deep learning architectures—including recurrent neural networks, temporal convolutional networks, and even attention-based transformers—have been found to struggle in efficiently modeling long sequences, owing to computational bottlenecks, limited memory capacity, and instability in gradient propagation. Moreover, these models typically require extensive labeled datasets and frequent retraining when deployed across different domains, such as in new cities or alternative sensor networks.

A promising direction for overcoming these limitations lies in the paradigm of pretraining, which has already transformed fields such as NLP. Pretraining refers to the process through which generalized representations are learned from large-scale, unlabeled data and subsequently fine-tuned on downstream tasks with minimal additional supervision. In NLP, models such as BERT and GPT achieve this by employing masked token prediction or next-token prediction to capture global dependencies across extensive text corpora. The central advantage of pretraining is that it decouples the representation learning phase from task-specific objectives, thereby allowing models to generalize more effectively and adapt more rapidly to new scenarios.

Motivated by this success, recent research in traffic prediction has begun to explore the transferability of pretraining strategies originally developed for NLP to the spatiotemporal domain. However, the direct transfer of pretraining strategies from NLP to traffic forecasting is far from straightforward, as traffic data introduces a distinct set of challenges that complicate such adaptation. Unlike words in natural language, which inherently carry semantic meaning and contextual associations, individual traffic data points lack intrinsic semantic richness and are highly vulnerable to stochastic fluctuations induced by external disturbances—such as weather anomalies, sensor failures, or traffic accidents. As a result, the extraction of stable and context-aware representations becomes substantially more difficult. Moreover, the temporal sequences observed in traffic datasets are typically much longer and more fine-grained than those encountered in linguistic corpora. While a sentence in NLP may consist of only tens of tokens, traffic time series often span thousands of time steps, thereby imposing significantly greater computational burdens during both training and inference phases. Most critically, effective traffic forecasting necessitates the joint modeling of spatial dependencies and temporal dynamics, a requirement that standard Transformer-based pretraining frameworks—originally developed for unstructured text—are not inherently designed to fulfill. These limitations collectively underscore the need for domain-specific innovations in both model architecture and pretraining methodology, in order to fully harness the potential of self-supervised learning for traffic prediction tasks.

To address these domain-specific constraints, recent approaches have incorporated SSM and DDPM into traffic prediction pipelines. These modeling paradigms have been shown to reduce computational complexity, improve robustness against noise, and naturally support masking-reconstruction tasks that are well-suited for pretraining. By leveraging the representational flexibility of SSM and the generative learning capabilities of DDPM, these approaches aim to build more adaptable, scalable, and accurate traffic forecasting systems.

#### Selective state space models

To address the limitations of Transformer-based models in capturing long-term dependenciesparticularly the high computational complexity associated with attention mechanisms $\:\left(\mathcal{O}\left({\text{L}}^{2}\right)\right)$ researchers have proposed SSM as a more efficient alternative for sequence modeling. An SSM characterizes the evolution of hidden states through a set of continuous-time, linear, time-invariant differential equations, which are naturally suited to dynamic systems such as traffic networks.

As outlined in a recent study^19^, the general form of an SSM can be expressed as:

1 $$\:\frac{\text{d}\text{h}\left(\text{t}\right)}{\text{d}\text{t}}=\text{A}\text{h}\left(\text{t}\right)+\text{B}\text{u}\left(\text{t}\right),\:\text{y}\left(\text{t}\right)=\text{C}\text{h}\left(\text{t}\right)+\text{D}\text{u}\left(\text{t}\right)$$

where $\:\text{u}\left(\text{t}\right)$ denotes external influences such as traffic signals or weather conditions. This formulation supports discrete-time update mechanisms, which preserve linear computational scaling with respect to sequence length^19^, thereby enabling the model to remain efficient even when applied to extended traffic sequences.

To further develop this framework, an analogy has been drawn by your work^28^ between SSM and attention mechanisms, demonstrating that linear regression in the state update process can be interpreted as a form of attention-where each prediction corresponds to a weighted sum of previous outputs:

2 $${\hat{\text{y}}} = \text{X}( \text{X}^{\text{T}}\text{X})^{ - 1} \text{X}^{\text{T}} \text{y}$$

This perspective provides theoretical support for replacing computationally intensive attention operations with more efficient state evolution functions. The Mamba module, as introduced in your paper^29^, builds upon this idea by incorporating selective mechanisms, analogous to dynamic gating, which allow the model to memorize and propagate relevant temporal signals while suppressing irrelevant ones. Such SSM offer several critical advantages that render them particularly well-suited for traffic forecasting applications. First, they facilitate the efficient modeling of long temporal sequences with linear computational complexity $\:\left(O\left(L\right)\right)$, in contrast to the quadratic scaling inherent in traditional attention mechanisms. This property allows SSM to scale effectively to traffic datasets that span multiple days or weeks at high temporal resolutions. Second, their selective gating mechanisms enable dynamic adaptation to both local and global spatiotemporal patterns. As a result, these models are capable of capturing transient phenomena, such as rush-hour surges, as well as longer-term periodic trends, including daily or weekly traffic cycles. Finally, as demonstrated in prior work^30^, such models naturally accommodate the integration of exogenous variables—including weather conditions, road events, and traffic signals—through additive residual pathways. This not only enhances the model’s flexibility in diverse real-world contexts but also improves its interpretability, which is crucial for operational deployment in intelligent transportation systems.

Crucially, selective SSM such as Mamba demonstrate strong performance without requiring multi-head attention layers, making them especially well-suited for low-latency, real-time forecasting in smart city applications.

#### Denoising diffusion probabilistic models

While SSM offer efficient modeling of sequence evolution, they typically assume that the input stream is relatively complete and well-conditioned. In contrast, real-world traffic data are often incomplete, corrupted, or subject to abrupt fluctuations. To enable robust learning under such noisy conditions, your study incorporates DDPM^28,29^. DDPM are generative models designed to learn data distributions through a two-phase process:

1. Forward diffusion, in which Gaussian noise is gradually added to the input data over multiple time steps.
2. Reverse denoising, in which the model learns to iteratively remove noise and recover the original clean signal.

**Fig. 3:**
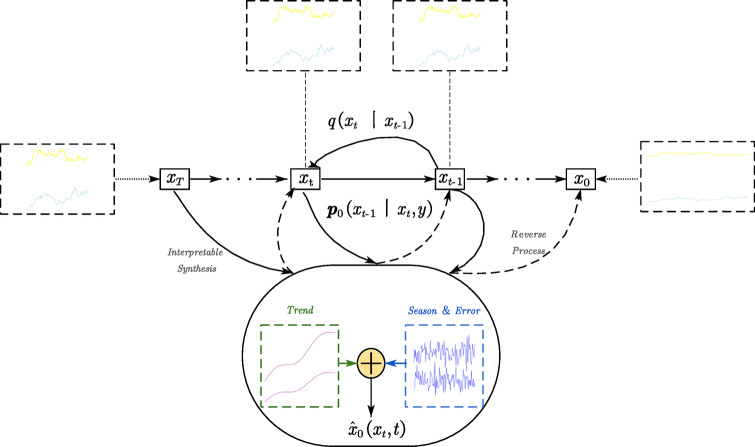
In diffusion models, time series data undergo both forward and reverse diffusion processes.

This two-phase process is illustrated in Fig. 3, which shows how a traffic signal is progressively corrupted by noise in the forward diffusion steps and then reconstructed during reverse denoising. The visualization helps convey how the model learns to restore underlying patterns through iterative refinement.

**Fig. 4:**
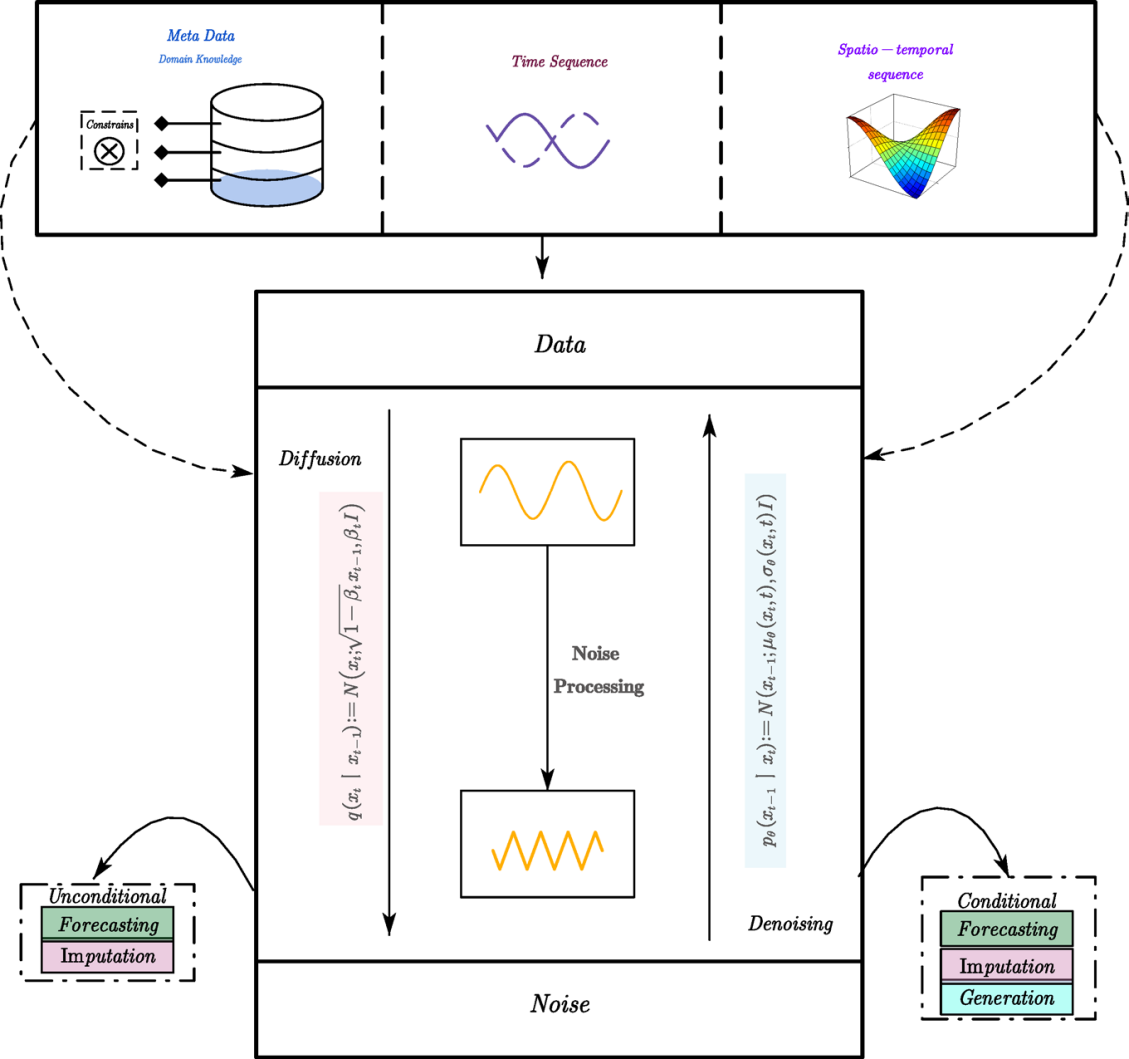
This study provides an overview of diffusion models for time series and spatiotemporal data analysis. In the diffusion process, $\:{x}_{t}$ and $\:{x}_{t-1}$ are denoted as the results after noise is added at steps $\:t$ and $\:t-1$, respectively. The process is represented by a control step $\:{\beta\:}_{t}\epsilon \left(\text{0,1}\right)$, an identity matrix $\:I$, and a Gaussian distribution $\:N\left(x,\mu\:,\sigma\:\right)$ over $\:x$ with mean $\:\mu\:$ and covariance $\:\sigma\:$. During the denoising process, the data distribution is iteratively learned by modeling $\:{p}_{\theta\:}\left({x}_{t-1}|{x}_{t}\right)$. The functions $\:{\mu\:}_{\theta\:}\left(\bullet\:\right)$ and $\:{\sigma\:}_{\theta\:}\left(\bullet\:\right)$ are treated as the model’s learnable parameters.

As described in^29^, the forward diffusion process can be formalized as:

3 $$\:\text{q}\left({\text{x}}_{\text{t}}\mid\:{\text{x}}_{\text{t}-1}\right)=\mathcal{N}\left({\text{x}}_{\text{t}};\sqrt{1-{{\upbeta\:}}_{\text{t}}}{\text{x}}_{\text{t}-1},{{\upbeta\:}}_{\text{t}}\text{I}\right)$$

where $\:{{\upbeta\:}}_{\text{t}}$ denotes a variance schedule that controls the noise level. In the reverse process, the model is trained to learn a posterior distribution $\:\text{p}\left({\text{x}}_{\text{t}-1}\mid\:{\text{x}}_{\text{t}}\right)$, parameterized by a neural network. As highlighted in your study^30,31^, DDPM are particularly well-suited for traffic prediction tasks due to their inherent ability to handle noisy and incomplete data through probabilistic generative modeling. First, they can reconstruct missing values by leveraging contextual information from surrounding observations, effectively treating corrupted inputs as noise-injected segments within the diffusion framework. This enables the model to maintain robust representations even in the presence of substantial sensor outages or anomalies. Second, DDPM are highly compatible with pretraining paradigms, as the forward diffusion process—where data is deliberately corrupted—closely mimics real-world scenarios involving incomplete or disrupted traffic data. By training the model to denoise these corrupted inputs, the system learns to infer latent spatiotemporal structure without requiring fully labeled data. These characteristics collectively make DDPM an effective backbone for robust and transferable representation learning in large-scale, noise-prone traffic forecasting applications. A deeper look into the spatiotemporal modeling capabilities of DDPM is provided in Fig. 4, which visualizes how the denoising process preserves both temporal progression and spatial coherence over traffic nodes, supporting structured recovery across the network.

**Fig. 5:**
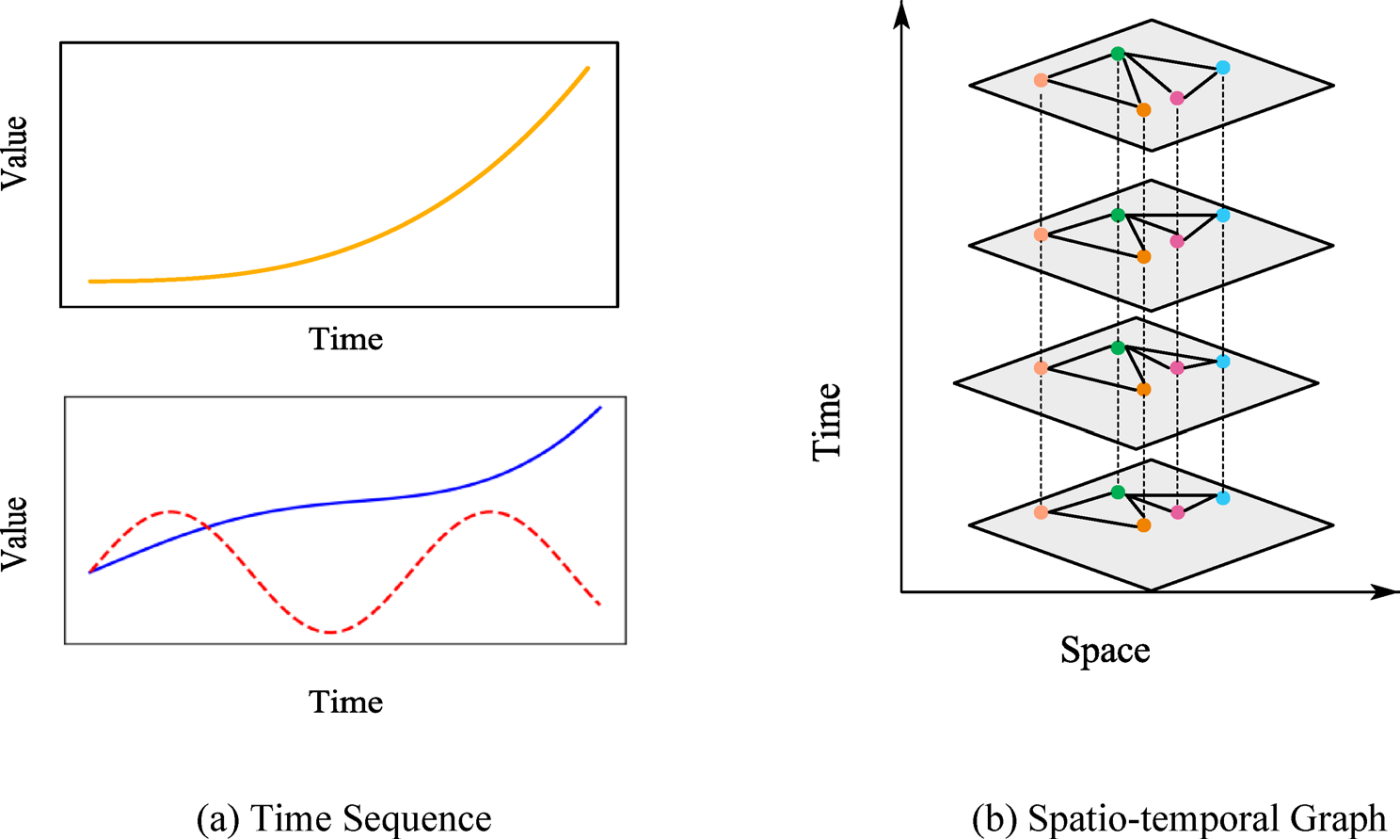
Different data sequence types.

To clarify the distinction between simple time series and structured spatiotemporal graph inputs, Fig. 5 compares traditional univariate and multivariate time series (5a) with graph-based representations of traffic flow (5b), highlighting how spatial relationships are explicitly encoded and preserved during the learning process. This process enables the model to establish strong priors over both short-term variability and long-term structure, even in the presence of significant input corruption. When integrated with the Mamba encoder, DDPM contributes to a multi-level representation: combining the structured temporal inference of SSM with the robustness afforded by generative modeling. Collectively, the integration of Mamba-based selective state space modeling and DDPM-based generative pretraining constitutes a complementary dual mechanism for spatiotemporal traffic forecasting. This architecture is capable of effectively learning from long, noise-prone traffic sequences by capturing both structural regularities and stochastic variations across temporal and spatial dimensions. Moreover, by decoupling representation learning from task-specific supervision, the proposed framework facilitates transfer learning across heterogeneous urban traffic datasets, thereby enabling adaptation to varying sensor distributions and network topologies. Additionally, the use of a pretrained backbone significantly reduces the retraining and adaptation burden typically required for model deployment in new environments, thus enhancing the scalability and real-world applicability of the system.

### Summary and positioning of our contribution

The evolution of traffic forecasting methodologies over recent decades reflects a progressive enhancement in the modeling of spatiotemporal dynamics. Early statistical and shallow learning models—such as ARIMA, KNN, and GRU^21–23^—provided lightweight solutions for short-term prediction tasks but lacked the expressive capacity necessary to capture the nonlinear and high-dimensional patterns characteristic of modern traffic systems. These limitations motivated the development of graph-based neural networks and attention-augmented architectures^23–26^, which introduced spatial awareness and dynamic temporal reasoning into forecasting frameworks. Nonetheless, despite these advancements, such models continue to exhibit several practical deficiencies, including sensitivity to missing data, inefficiencies in handling long temporal sequences, and poor generalization across urban contexts with differing topologies or sensor configurations.

In response to these enduring challenges, the research community has increasingly turned to pretraining paradigms inspired by advances in NLP^27^, with the aim of decoupling representation learning from downstream forecasting objectives. However, as previously discussed, traffic data presents a set of unique complexities—including limited semantic granularity, high temporal resolution, and the necessity of jointly modeling spatial and temporal dependencies—which are not readily addressed by standard pretraining methods designed for unstructured text data. These domain-specific constraints necessitate the development of tailored architectural and training innovations, rather than direct adaptations of NLP-based frameworks.

To address this need, we propose a unified modeling framework that integrates two complementary strategies: SSM and DDPM^19,28–31^. The SSM component, implemented using the Mamba architecture^29,30^, enables efficient long-range sequence modeling by dynamically adapting to multi-scale spatiotemporal patterns. This allows the encoder to process long, fine-grained traffic sequences with linear time complexity, thereby avoiding the quadratic computational overhead characteristic of attention-based models. Concurrently, the DDPM module introduces a generative pretraining strategy that is specifically tailored to the properties of traffic data. By treating masked or incomplete inputs as noise-injected segments, DDPM facilitates the reconstruction of missing values and the recovery of global structure through a progressive denoising process^28–31^.

These two components collectively form the foundation of the ImPreSTDG framework, which is designed to address three critical limitations observed in current traffic forecasting research:

1. A limited capacity to model long-term dependencies without incurring exponential growth in computational complexity;
2. Insufficient robustness to noisy or incomplete data, particularly in scenarios involving sensor failure or environmental disruption;
3. Poor cross-domain generalization, which renders model transfer across cities or networks computationally expensive and operationally inefficient.

By pretraining a scalable, robust, and transferable spatiotemporal encoder, the proposed framework enables rapid adaptation to diverse downstream forecasting tasks with minimal fine-tuning. Extensive experiments conducted on multiple real-world traffic datasets validate the effectiveness of this approach, demonstrating consistent improvements in both predictive accuracy and computational efficiency when compared to existing state-of-the-art models.

## Method

### Traffic network definition

In this study, the road network is defined as $\:G=\left(N,D\right)$, where $\:N=\left\{{n}_{1},{n}_{2},\cdots\:,{n}_{K}\right\}$ denotes the set of nodes, with each loop detector treated as a node. The number of nodes is represented by $\:K$.$\:D$ represents the set of edges, where $\:{d}_{i,j}$ indicates the connection between two nodes. $\:X\in\:{\mathbb{R}}^{K\times\:L}$ denotes the historical traffic data of $\:K$ nodes over a historical period $\:L$. Assuming the current time is $\:t$, the traffic forecasting problem is formulated to learn the mapping function $\:f$, which predicts future traffic data for $\:\text{P}$ steps using the past $\:L$ historical data:

4 $$\:\left[{\widehat{X}}_{t+1},\cdots\:,{\widehat{X}}_{t+P}\right]=f\left(G;\left[{X}_{t-L+1},\cdots\:,{X}_{t}\right]\right)$$

where $\:\left[{\widehat{X}}_{t+1},\cdots\:,{\widehat{X}}_{t+P}\right]\in\:{\mathbb{R}}^{K\times\:P}$ represents the predicted values, and $\:\left[{X}_{t-L+1},\cdots\:,{X}_{t}\right]\in\:{\mathbb{R}}^{K\times\:L}$ represents the historical data. $\:{\widehat{\:X}}_{i}$ denotes the predicted value, while $\:{X}_{i}$ denotes the actual traffic data at node $\:i$.

### Graph convolutional network

The GCN model is a powerful tool used on graph-structured data. The connectivity of roads is represented by an adjacency matrix, capturing the topology features of road networks. The $\:l$th layer of the GCN model is defined as $\:{Q}^{\left(l\right)}=\left[{q}_{1}^{\left(l\right)},\cdots\:,{q}_{K}^{\left(l\right)}\right]\in\:{\mathbb{R}}^{K\times\:{O}^{\left(l\right)}}$ and $\:i\in\:K$, where $\:{q}_{i}^{\left(l\right)}$ represents node $\:i$ with a length of $\:{Q}^{\left(l\right)}$.$\:\:{Q}^{\left(l\right)}$ is the dimension of the $\:l$th hidden layer, and if $\:M$ is the last layer, $\:{Q}^{\left(L\right)}=P$. For convenience, $\:X$ is denoted as $\:{Q}^{\left(0\right)}$. The adjacency matrix $\:A\:\in\:\:{\mathbb{R}}^{K\times\:K}$ contains values of 0 or 1, where 0 indicates no connection between nodes and 1 indicates a connection. $\:{E}_{i,i}={\sum\:}_{j}A\in\:\:{\mathbb{R}}^{K\times\:K}$ represents the diagonal degree matrix of $\:A$. As defined by Kipf and Welling^31^, the$\:\:(l\:+\:1)$th convolutional layer is:

5 $$\:{q}_{i}^{(l+1)}=\theta\:\left(\sum\:_{j\in\:sn\left(i\right)}\frac{1}{\sqrt{{\stackrel{\sim}{E}}_{i,i}{\stackrel{\sim}{E}}_{j,j}}}{q}_{j}^{\left(l\right)}{W}^{\left(l\right)}\right)$$

where $\:Kn\left(i\right)$ denotes node $\:n\left(i\right)$ and its neighbors.

The equivalent matrix form is given as follows:

6 $$\:{Q}^{(l+1)}=\theta\:\left({\stackrel{\sim}{E}}^{-\frac{1}{2}}\stackrel{\sim}{A}{\stackrel{\sim}{E}}^{-\frac{1}{2}}{Q}^{\left(L\right)}{W}^{\left(l\right)}\right)$$

where$\:\:\stackrel{\sim}{A}\:=\:A\:+\:{I}_{K},\:\stackrel{\sim}{E}\:=\:E\:+\:{I}_{K}$, and $\:I$ is the identity matrix. $\:{Q}^{(l+1)}\:\in\:{\mathbb{R}}^{K\times\:{O}^{(l+1)}}\:\:$represents the output of the$\:\:(l\:+\:1)$th layer, $\:W\left(l\right)\:\in\:\:RQ\left(l\right)\times\:Q(l+1)$ denotes the weight parameters, and $\:\theta\:$ is the nonlinear activation function.

### Temporal attention layers

The aim of this module is to estimate the saliency and relevance of each sequence observation. The saliency score should be based not only on the current time step’s input but also on information from neighboring observations in both directions.

7 $$\:{a}_{t}=\sigma\:\left({m}^{\top\:}\left({\overrightarrow{h}}_{t};{\overleftarrow{h}}_{t}\right)+b\right)$$

where, $\:\text{m}$ is the weight vector of the fusion layer that integrates both directional layers, and $\:b$ is the bias term. A sigmoid function $\:{\upsigma\:}$ is employed as the activation function at the top layer of the attention module in Eq. (7) to constrain the attention weight to lie between $\:\left[\text{0,1}\right]$.

The vectors $\:{\overrightarrow{\text{h}}}_{\text{t}}$ and $\:{\overleftarrow{\text{h}}}_{\text{t}}$ are the hidden representations of module:

8 $$\:{\overrightarrow{\text{h}}}_{\text{t}}=\text{g}\left(\overrightarrow{\text{W}}{\text{x}}_{\text{t}}+\overrightarrow{\text{U}}{\overrightarrow{\text{h}}}_{\text{t}-1}+\overrightarrow{\text{b}}\right)$$

9 $$\:{\overleftarrow{\text{h}}}_{\text{t}}=\text{g}\left(\overleftarrow{\text{W}}{\text{x}}_{\text{t}}+\overleftarrow{\text{U}}{\overleftarrow{\text{h}}}_{\text{t}+1}+\overleftarrow{\text{b}}\right)$$

ReLU functions are used as the activation functions $\:g$.

Furthermore, another important role the learned attention weights play is to provide interpretability regarding the degree of salience of each time step.

### Pre-training framework

The pre-training model proposed in this study offers several significant advantages. (1) **Denoising Process Enhances Stability**: The denoising process stabilizes the model’s ability to handle random fluctuations and noise in the data. By utilizing a denoising probabilistic model, effective representations are learned from noisy data through forward diffusion and reverse denoising, improving the model’s capacity to manage noise and missing data. This approach is especially effective in addressing the common issues of noise and missing data in traffic forecasting, such as sensor failures or environmental factors. (2) **Integrating long-term and short-term historical data enhances forecasting capabilities**: By employing pre-training and fine-tuning, the proposed method extracts global features from long-term historical data and integrates short-term local trends, enabling comprehensive modeling of complex spatio-temporal dependencies and significantly enhancing the accuracy and robustness of traffic forecastings. (3) **Versatility and rapid deployment**: The backbone network generated through pre-training is highly versatile, capable of integrating with various prediction heads to adapt to a wide range of downstream tasks. Moreover, pre-trained models can be transferred across datasets, substantially reducing retraining requirements, expediting deployment, and enhancing efficiency. (4) **Efficient long-term spatiotemporal modeling**: The Mamba encoder employs a selective state-space model, crucial for capturing long-term dependencies in traffic data.

**Fig. 6:**
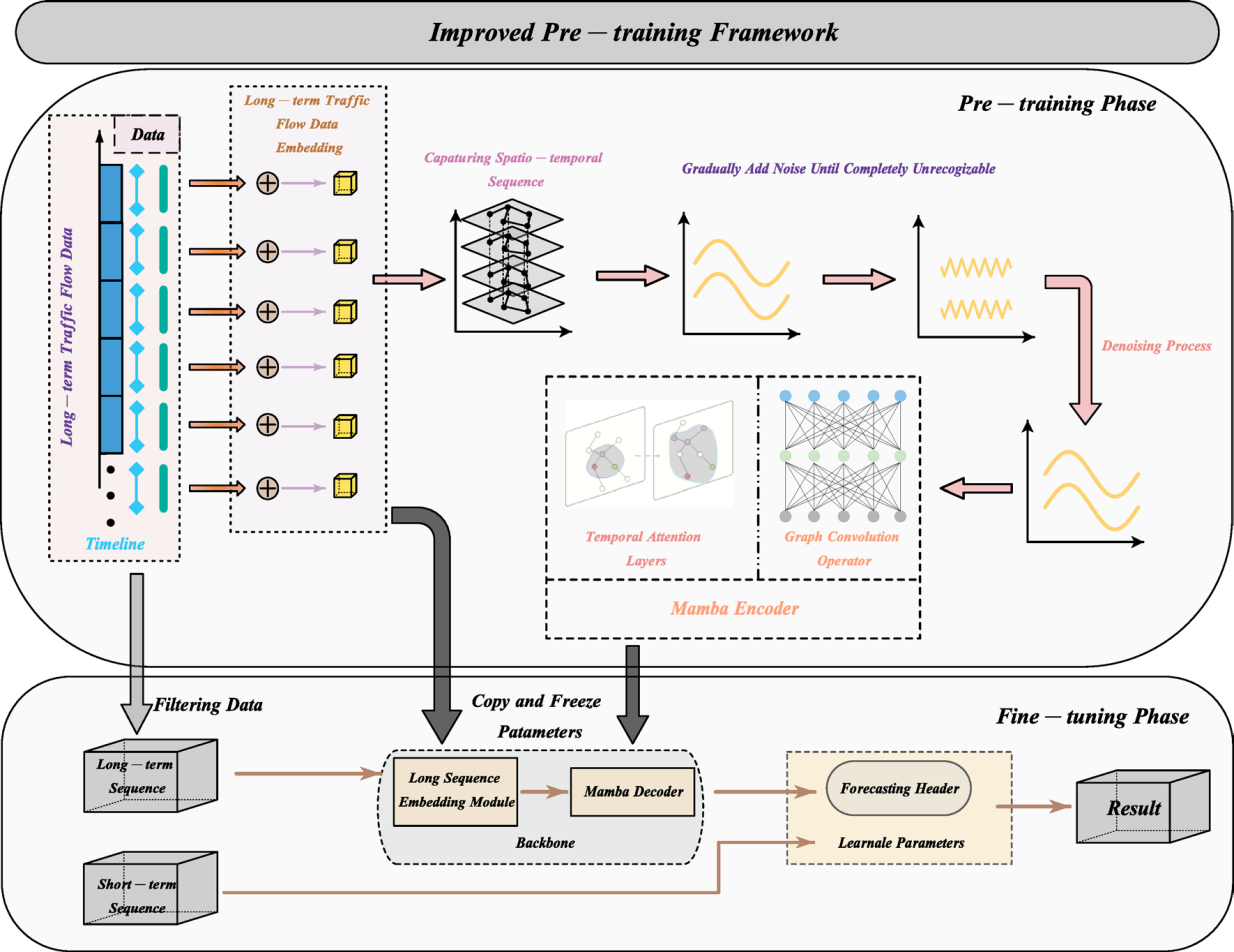
The improved pre-training framework shown in this study.

Building upon the preceding discussion, the complete architecture of the proposed traffic forecasting framework—ImPreSTDG is depicted in Fig. 6. The model is constructed as a modular pipeline that sequentially integrates three core components: graph-based spatial modeling, state-space-based temporal encoding, and diffusion-based generative pretraining, culminating in a lightweight forecasting head. Each module contributes a distinct function within the overall learning framework and is formally defined in the subsequent sections.

1. Spatial Encoder

The raw input sequence $\:\text{X}\in\:{\mathbb{R}}^{\text{N}\times\:\text{T}\times\:\text{F}}$, where $\:\text{N}$ denotes the number of nodes, $\:\text{T}$ represents the number of observed time steps, and $\:\text{F}$ is the number of input features, is initially processed through a graph convolutional encoder. Spatial dependencies among nodes are captured using a normalized adjacency matrix $\stackrel{\sim}{A}$. At each time step $\:\text{t}$, spatial representations $\:{\text{H}}_{\text{t}}\in\:{\mathbb{R}}^{\text{N}\times\:\text{D}}$ are computed as follows:

10 $$\:{\text{H}}_{\text{t}}={\upsigma\:}\left(\stackrel{\sim}{A}{\text{X}}_{\text{t}}{\text{W}}_{1}+{\text{b}}_{1}\right)$$

where $\:{\text{W}}_{1}$ is a learnable weight matrix and $\:{\upsigma\:}$ denotes a nonlinear activation function. The output is a tensor $\:\text{H}\in\:{\mathbb{R}}^{\text{N}\times\:\text{T}\times\:\text{D}}$, which encodes localized spatial features across the sequence.

2. Temporal Encoder

To capture long-range temporal dependencies, we utilize a SSM instantiated via the Mamba architecture. The spatial features $\:\text{H}$ are fed into the SSM module, which performs efficient, linear-time sequence modeling over the temporal axis:

11 $$\:\text{Z}=\text{S}\text{S}\text{M}\left(\text{H}\right)\in\:{\mathbb{R}}^{\text{N}\times\:\text{T}\times\:\text{D}}$$

The Mamba kernel dynamically selects and aggregates information across time steps through structured state transitions, enabling the model to efficiently encode both short-term variations and long-term temporal patterns.

3. Pretraining Decoder

In parallel with supervised learning, a DDPM is incorporated to enable self-supervised pretraining. During this phase, the input $\:\text{X}$ is partially masked to obtain $\:\overline{\text{X}}$, which is then corrupted via a forward Gaussian diffusion process:

12 $$\:{\overline{\text{X}}}_{\text{t}}=\sqrt{1-{{\upbeta\:}}_{\text{t}}}{\overline{\text{X}}}_{\text{t}-1}+{\epsilon}_{\text{t}},\:{\epsilon}_{\text{t}}\sim\:\mathcal{N}\left(0,{{\upbeta\:}}_{\text{t}}\text{I}\right)$$

The DDPM decoder is trained to reverse this noise injection via a denoising process:

13 $${\hat{\text{X}}} =\epsilon_{{\uptheta\:}}\left({\overline{\text{X}}}_{\text{t}},\text{t}\right)$$

This results in a pre-trained representation $\:{\text{Z}}_{\text{D}\text{D}\text{P}\text{M}}\in\:{\mathbb{R}}^{\text{N}\times\:\text{T}\times\:\text{D}}$, which aligns with the masked input regions and supports both missing data reconstruction and regularization of the supervised training process.

4. Forecasting Head

The outputs from the SSM-based temporal encoder and the DDPM decoder are concatenated and passed into a Meta-Fusion Block, which consists of either a lightweight 1D convolutional layer or a spatiotemporal attention module, depending on the task requirements. The final prediction is computed as:

14 $${\hat{\text{Y}}}=\text{M}\text{e}\text{t}\text{a}\text{F}\text{u}\text{s}\text{i}\text{o}\text{n}\left(\text{Z},{\text{Z}}_{\text{D}\text{D}\text{P}\text{M}}\right)\in\:{\mathbb{R}}^{\text{N}\times\:{\text{T}}^{{\prime\:}}\times\:{\text{F}}^{{\prime\:}}}$$

where $\:{\text{T}}^{{\prime\:}}$ denotes the forecasting horizon and $\:{\text{F}}^{{\prime\:}}$ the number of target features.

This modular architecture supports end-to-end training while also enabling the decoupling of the pretraining and fine-tuning stages. The DDPM module can be independently trained using a masked reconstruction loss and subsequently frozen during downstream forecasting. The Mambabased SSM ensures efficient long-sequence temporal modeling, even across hundreds of time steps, while the GCN module maintains spatial consistency aligned with the underlying network topology.

## Experiment result and analysis

### Dataset

The road traffic flow data from Linyi, Qingdao and Jinan, collected between May 1 and December 1, 2018, is used in this study. This dataset is divided into three parts: IAJ, MAJ, and MMJ. The missing values in the dataset have been preprocessed by linear interpolation to fill in the missing values. Where, 70% is used as a training set, 20% as a test set, and 10% as a validation set. To evaluate the generalization ability of the model, three public traffic datasets PeMS03, PeMS04 and PeMS07 from the PeMS system of the California Department of Transportation were selected.

### Evaluation metric

Three metrics, Mean Absolute Error (MAE), Root Mean Square Error (RMSE) and Mean Absolute Percentage Error (MAPE), are introduced to measure the model’s performance in terms of prediction accuracy.

15 $$\:\text{M}\text{A}\text{E}=\frac{1}{KN}\sum_{t=1}^{K}\:\:\sum_{i=1}^{N}\:\:\left|{X}_{i\left(t\right)}-{f}_{i}\left(A,\stackrel{\sim}{X}_{t-\text{L}+1:t}\right)\right|$$

16 $$\:\text{R}\text{M}\text{S}\text{E}=\sqrt{\frac{1}{KN}\sum_{t=1}^{K}\:\:\sum_{i=1}^{N}\:\:{\left({X}_{i\left(t\right)}-{f}_{i}\left(A,\stackrel{\sim}{X}_{t-\text{L}+1:t}\right)\right)}^{2}}$$

17 $$\:\text{M}\text{A}\text{P}\text{E}\:=\frac{1}{\text{K}\text{N}}\sum_{\text{t}=1}^{\text{K}}\sum_{\text{i}=1}^{\text{N}}\left|\frac{{\text{X}}_{\text{i}\left(\text{t}\right)}-{\text{f}}_{\text{i}}\left(\text{A},\stackrel{\sim}{X}_{\text{t}-\text{L}+1:\text{t}}\right)}{{\text{X}}_{\text{i}\left(\text{t}\right)}}\right|$$

Task performance is assessed by reporting MAE, RMSE and MAPE values based on the unperturbed input. In this study, MAE, RMSE and MAPE are employed to reflect the model’s performance.

### Experiment setting

#### Models for comparison

In the comparative experiment, several state-of-the-art models and algorithms are used to compare with the proposed model.

- **MDGCRN (Multi-scale Fusion Dynamic Graph Convolutional Recurrent Network)**^32^: MDGCRN is a technique for creating dynamic graphs, which can effectively capture both short-term and long-term temporal information, as well as spatial information, when combined with GRU.
- **STHGFormer (Spatio-Temporal Heterogeneous Graph Transformer)**^33^: STHGFormer incorporates a Heterogeneous Spatial Embedding (HSE) module to encode road network information, including diverse attributes and interactions within Heterogeneous Road network Graph (HRG). Based on the spatial information encoded by HSE, the unified SpaFormer serves as the spatial module of STHGFormer to capture the interdependencies of roads across the entire traffic network. An Adaptive Soft Threshold (AST) module is embedded in TempFormer, enabling dynamic threshold adjustment to enhance the ability to analyze complex temporal correlations.
- **FEHGCARN (Fourier-enhanced heterogeneous graph convolution attention recurrent network)**^34^: FEHGCARN integrates the Graph Convolutional Attention Recurrent Unit (GCARU) with a Fourier-enhanced heterogeneous graph learning module, facilitating the capture of complex relationships between nodes in the frequency domain.
- **Multi-level Graph Memory Network Cluster Convolutional Recurrent Network (MMNCCRN)**^35^: MMNCCRN comprises four modules: the Encoder Module (EM), Attention Module (AM), Memory Network Cluster Module (MNCM), and Decoder Module (DM). Additionally, a memory network is employed, and clustering is incorporated. By storing and memorizing patterns embedded in spatial and temporal dependencies, it aids the model in learning the underlying graph structure and discovering new hidden patterns.
- **Spatial-Temporal Selective State Space Synchronization (ST-MambaSync)**^36^: ST-MambaSync integrates Mamba and Transformer.
- **Spatial-Temporal Selective State Space (ST-Mamba)**^37^: ST-Mamba integrates an efficient Spatiotemporal Mixer (ST-Mixer) to seamlessly combine spatial and temporal data processing into a unified framework, while employing a Spatiotemporal Selective State-space (ST-SSM) block to enhance computational efficiency.

#### Hyperparameter settings

During the pre-training phase, the hyperparameters for long sequence embedding are set as follows: $\:\text{L}=7,\text{D}=64.\text{M}={T}_{l}/\text{L}$ is determined by the length of 3 days of data. All hidden feature dimensions are set to $\:\text{F}=\text{F}=64$. Optimization is performed using the Adam optimizer, starting with a learning rate of 0.001 that gradually decreased.

During the fine-tuning phase, all hidden dimensions are set to 64, and the kernel size of the spatialtemporal convolutions is set to 3. The dilation rates are $\:[\text{1,2},4]$, respectively.

### Experiment result

**Fig. 7:**
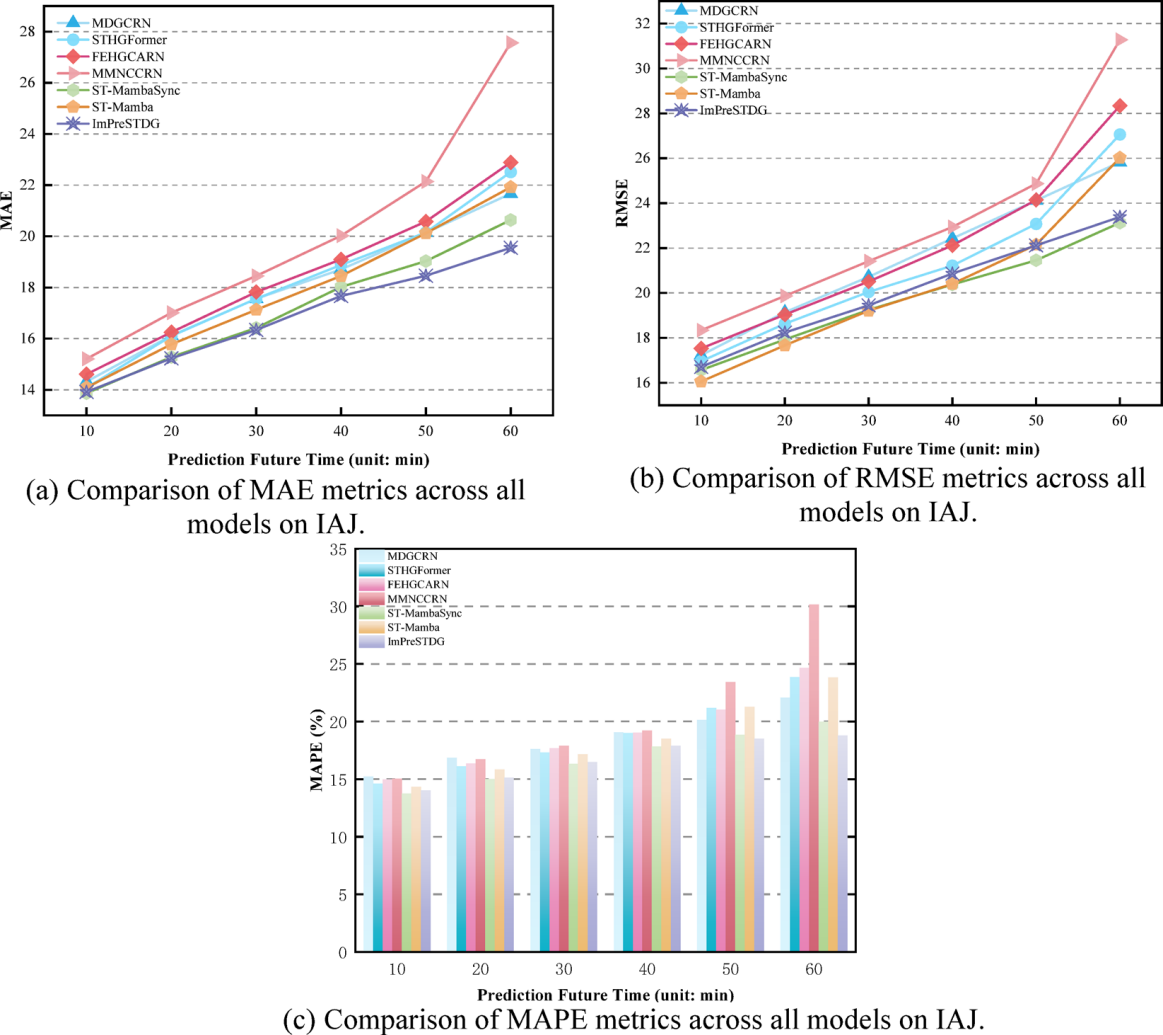
Comparison of performance metrics across all models on IAJ.

**Fig. 8:**
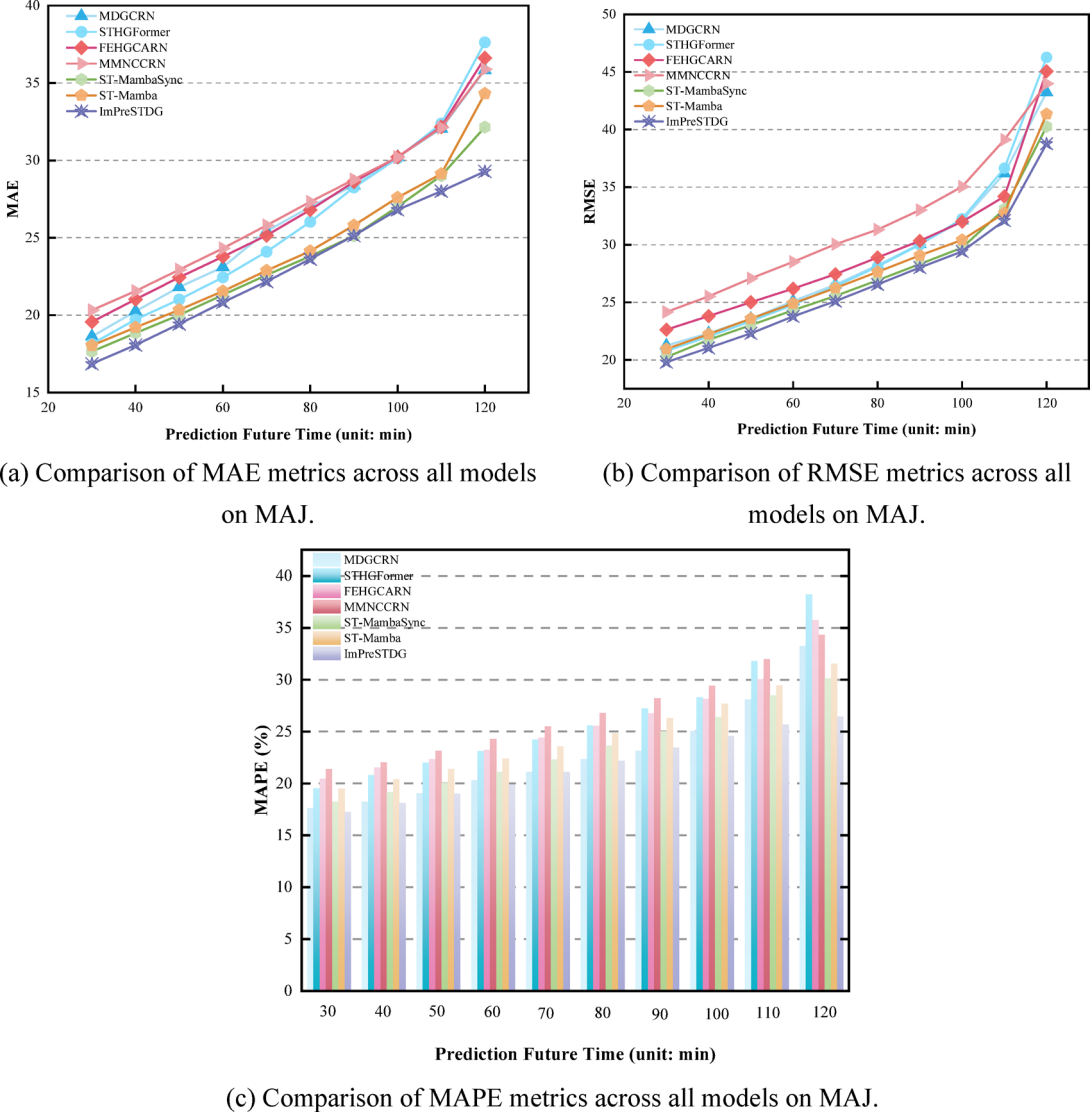
Comparison of performance metrics across all models on MAJ.

**Fig. 9:**
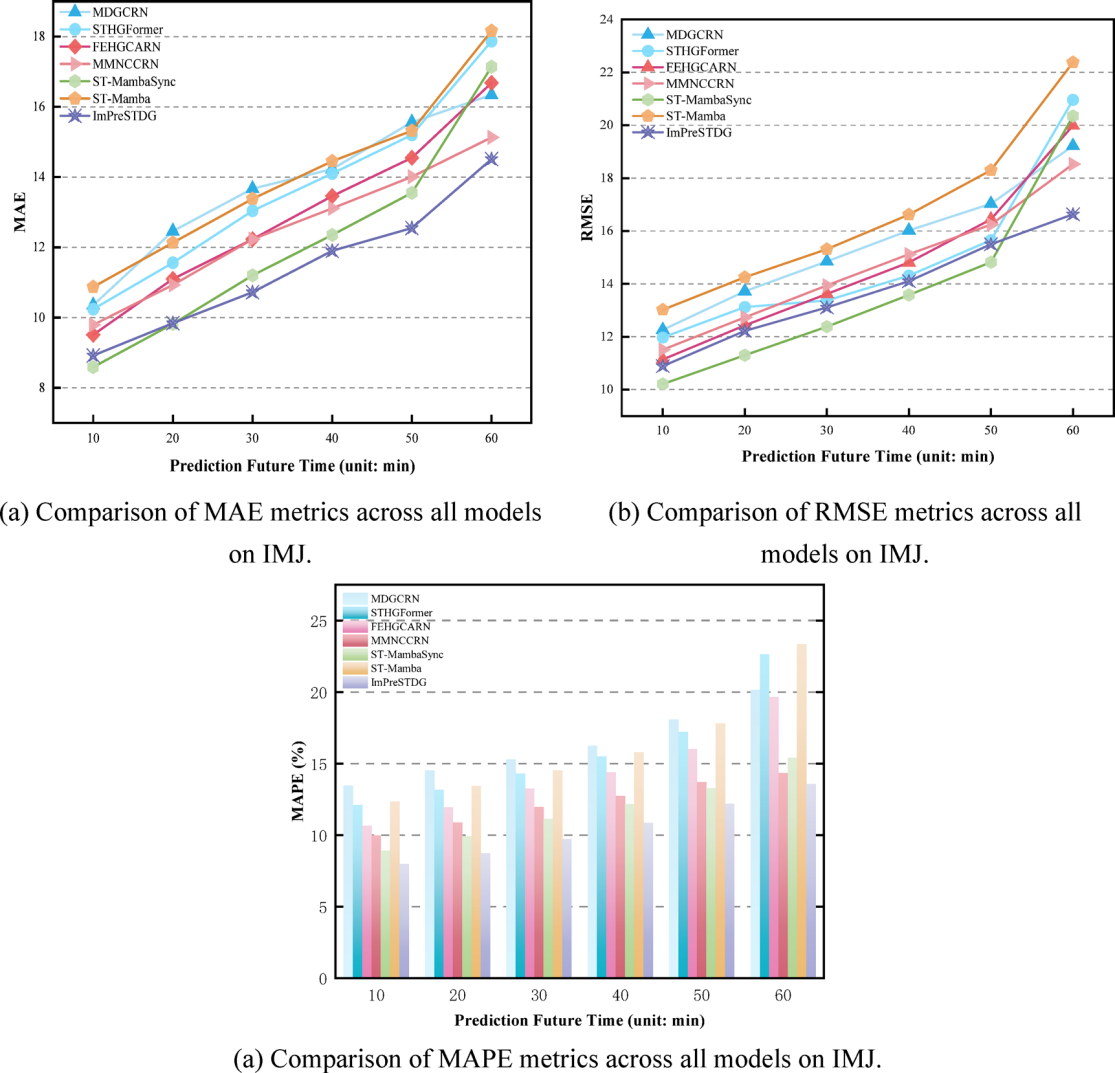
Comparison of performance metrics across all models on IMJ.

Tables 1, 2 and 3 show the prediction performance at different time steps across three datasets, with results reported for three time ranges for clarity. As shown in Tables 1, 2 and 3, the proposed ImPreSTDG outperforms all other methods on the three datasets, overcoming the limitations of focusing solely on short-term spatiotemporal relationships and effectively capturing long-term spatiotemporal semantics. Figs. 7, 8 and 9 visually illustrates the performance on the IAJ, MAJ and IMJ datasets.

In contrast, other models exhibit clear disadvantages. STHGFormer incorporates several complex modules, which could increase the model’s complexity and lead to high computational resource consumption during training and inference. Despite its strong performance on the Wuhan real-world dataset, it remains uncertain whether the model will perform similarly well in other cities or across different types of traffic networks. Although MDGCRN captures spatiotemporal dependencies via dynamic graph generation, it remains highly dependent on the dynamic graph construction process. If the dynamic graph generation process is inaccurate or unable to fully adapt to the highly dynamic traffic patterns, the accuracy of the results could be compromised. FEHGCARN depends on high-quality traffic datasets to extract meaningful spatiotemporal dependencies. If the data contains noise or missing values, the model may fail to fully capture the complex spatiotemporal relationships, potentially impacting prediction accuracy. Although FEHGCARN performs well at identifying sudden traffic events, it may still have limitations in handling high-frequency, short-term traffic fluctuations, such as those caused by sudden accidents or temporary traffic controls. The memory network is a key component of MMNCCRN, enabling the model to learn graph structures by storing and retaining patterns of spatial and temporal dependencies. However, the storage capacity and update mechanism of the memory network can become bottlenecks, particularly when handling very large datasets. As the model undergoes long-term training, the memory module may encounter overload issues, negatively impacting the model’s performance and efficiency. Although ST-MambaSync enhances prediction performance by integrating the Mamba model with the Transformer, its complex architecture—incorporating multiple levels of attention mechanisms and Mamba layers—may lead to significant resource consumption, particularly when processing large-scale datasets. This could result in memory bottlenecks or prolonged computation times, negatively impacting real-time application efficiency. ST-Mamba performs well in short-term forecasting, but both ST-Mamba and STAEformer exhibit limitations in long-term forecasting, such as 24-hour predictions. During periods of significant traffic fluctuations, such as rush hours or emergencies, the model’s predictions may diverge from actual traffic flow, suggesting that further optimization is needed to handle extreme fluctuations and long-term forecasts.

**Fig. 10:**
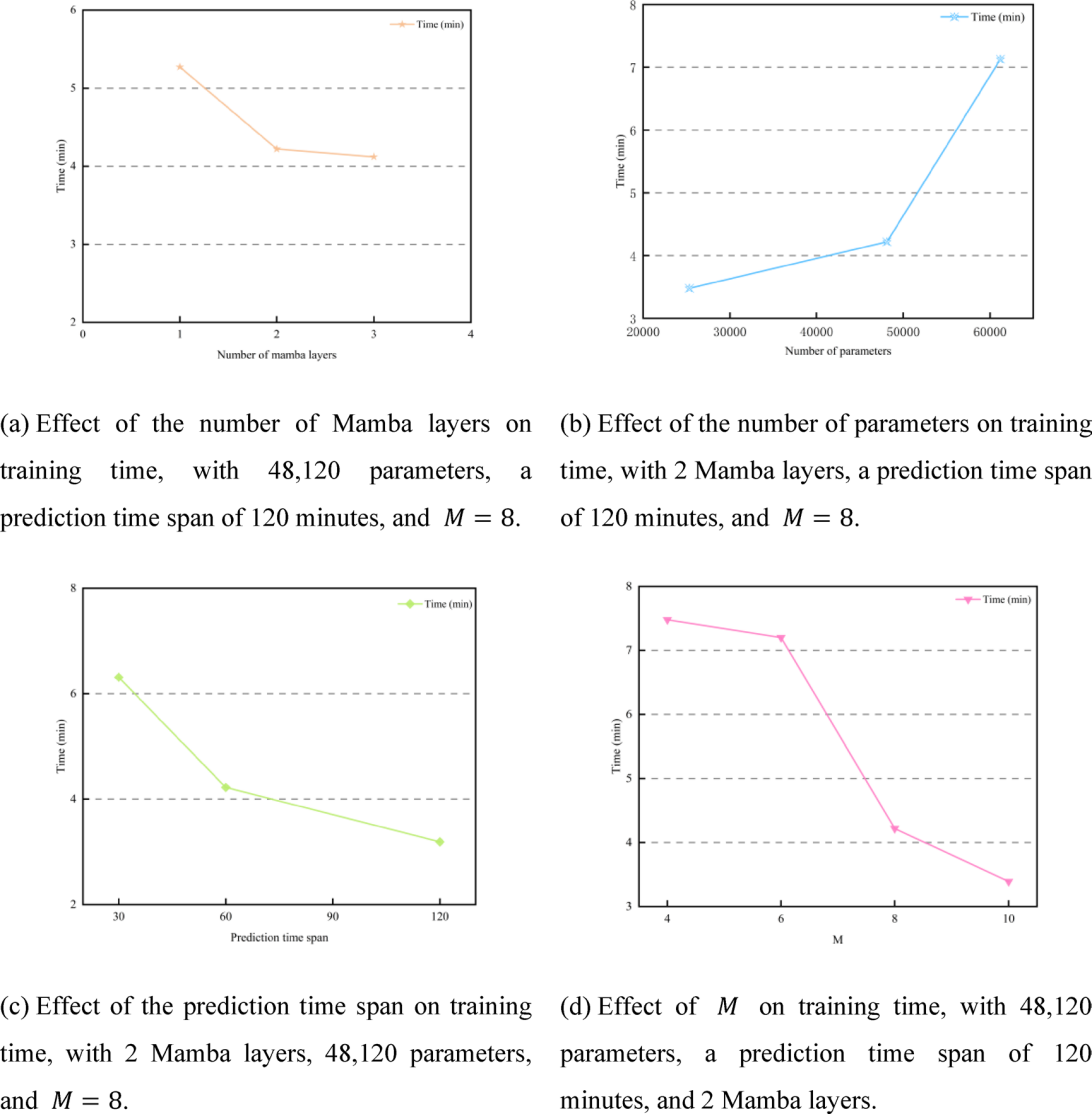
Training time on the MAJ dataset.

**Fig. 11:**
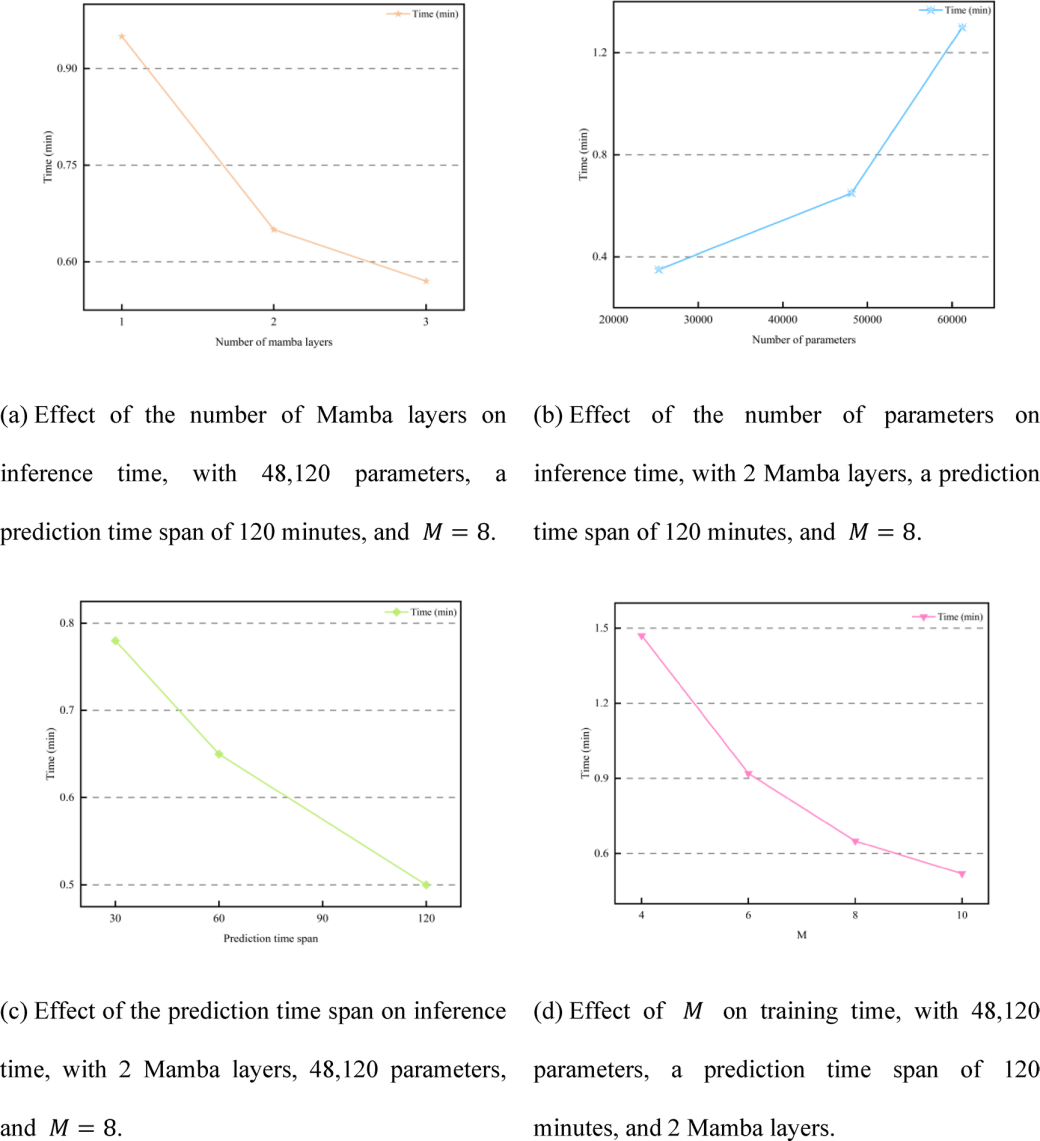
Inference time on the MAJ dataset.

Although ImPreSTDG achieved strong performance across the three indicators on the IAJ and IMJ datasets, there is a slight gap compared to other models in short-term prediction. This is because ImPreSTDG is designed to prioritize capturing long-term spatiotemporal dependencies, whereas in datasets like IAJ and IMJ, capturing short-term dependencies is more crucial. As a result, models such as STHGFormer and FEHGCAREN outperform ImPreSTDG in short-term predictions. However, despite being slightly lower in some short-term prediction indicators, ImPreSTDG demonstrates clear advantages in computational efficiency and model stability (as shown in Figs. 10, 11 and 7). By incorporating the Mamba module, the model significantly reduces computational costs when processing long-term traffic data, particularly in scenarios involving missing data and substantial environmental changes, while maintaining high prediction stability.

Since long-term traffic data requires processing hundreds or thousands of historical records, the computational complexity becomes $\:\text{O}\left({\text{T}}^{2}\right)$, which poses a significant computational burden. To address this issue, this paper proposes a long sequence embedding module that effectively reduces the computational pressure by lowering the complexity from $\:\text{O}\left({\text{T}}^{2}\right)$ to $\:\text{O}\left({\text{M}}^{2}\right)$, where $\:M$ is an adjustable parameter. As shown in Figs. 10 and 11, the control variable method is used to plot the training and inference times on the MAJ dataset. Regarding the number of Mamba layers, the optimal configuration is achieved when the number of layers is set to 2. Increasing the number of layers beyond this point leads to excessive computations and longer training times without a significant improvement in accuracy. Moreover, this increase in layers also raises the computational cost and may result in overfitting. In this study, $\:M$ is an adjustable parameter that controls the computational complexity of the long sequence embedding module. Specifically, $\:M$ represents the number of subsequences into which the long sequence data is divided. A larger value of $\:M$ reduces the length of each subsequence, lowering computational complexity but weakening the time dependency that can be captured. Conversely, a smaller value of $\:M$ increases the length of each subsequence, allowing for better capture of global time dependencies but at the cost of higher computational complexity. To balance the need for reduced computational complexity while capturing global time dependencies as effectively as possible, the final setting is $\:M=8$.

To evaluate the generalization ability of the model, three public traffic datasets PeMS03, PeMS04 and PeMS07 from the PeMS system of the California Department of Transportation were selected. When the prediction time spans are set to 60 min and 120 min, the results for three evaluation metrics MAE, RMSE, and MAPE are reported in Tables 4, 5 and 6, demonstrating the superiority of the proposed method.

**Table 1:** Prediction performance across different time steps on IAJ.

Models	IAJ (10 min/40 min/60 min)								
	MAE			RMSE			MAPE (%)		
MDGCRN	14.32	18.71	21.67	17.23	22.43	25.83	15.23	19.07	22.08
STHGFormer	14.08	18.88	22.51	16.96	21.22	27.06	14.59	19.01	23.86
FEHGCARN	14.61	19.10	22.89	17.53	22.12	28.34	14.97	19.04	24.67
MMNCCRN	15.21	20.02	27.56	18.34	22.94	31.28	15.03	19.22	30.17
ST-MambaSync	13.87	18.02	20.64	16.57	20.38	23.13	13.74	17.82	19.97
ST-Mamba	14.09	18.45	21.92	16.06	20.41	26.02	14.33	18.52	23.83
ImPreSTDG	13.92	17.67	19.55	16.71	20.87	23.39	14.01	17.89	18.79

**Table 2:** Prediction performance across different time steps on MAJ.

Models	MAJ (30 min/60 min/120 min)								
	MAE			RMSE			MAPE (%)		
MDGCRN	18.62	23.11	35.83	21.25	25.11	43.24	17.62	20.30	33.24
STHGFormer	18.14	22.44	37.62	20.79	24.78	46.27	19.54	23.10	38.21
FEHGCARN	19.57	23.78	36.62	22.63	26.19	45.05	20.45	23.24	35.75
MMNCCRN	20.32	24.32	35.88	24.17	28.52	43.99	21.37	24.30	34.34
ST-MambaSync	17.65	21.31	32.15	20.25	24.31	40.26	18.24	21.12	30.13
ST-Mamba	18.04	21.55	34.31	20.96	24.91	41.35	19.51	22.42	31.54
ImPreSTDG	16.86	20.83	29.28	19.81	23.78	38.77	17.24	19.90	26.44

**Table 3:** Prediction performance across different time steps on IMJ.

Models	IMJ (10 min/40 min/60 min)								
	MAE			RMSE			MAPE (%)		
MDGCRN	10.35	14.23	16.34	12.26	16.02	19.22	13.46	16.25	20.17
STHGFormer	10.24	14.10	17.87	11.97	14.31	20.96	12.11	15.50	22.64
FEHGCARN	9.51	13.46	16.68	11.14	14.81	19.99	10.65	14.38	19.66
MMNCCRN	9.79	13.11	15.13	11.51	15.12	18.53	9.97	12.74	14.34
ST-MambaSync	8.59	12.35	17.14	10.21	13.58	20.35	8.91	12.17	15.41
ST-Mamba	10.87	14.45	18.16	13.02	16.62	22.38	12.35	15.79	23.34
ImPreSTDG	8.92	11.90	14.51	10.89	14.10	16.63	7.99	10.85	13.57

**Table 4:** Prediction performance across different time steps on PeMS03.

Models	PeMS03 (10 min/40 min/60 min)								
	MAE			RMSE			MAPE (%)		
MDGCRN	30.79	35.87	41.19	34.67	39.26	46.32	12.27	14.07	13.17
STHGFormer	30.13	34.73	40.03	34.19	39.29	45.39	11.63	10.23	12.33
FEHGCARN	31.23	37.13	41.23	35.03	39.43	46.93	13.36	14.06	13.36
MMNCCRN	31.91	37.31	42.81	35.57	40.47	47.77	14.92	13.32	12.52
ST-MambaSync	29.57	34.37	39.37	33.42	38.72	44.12	9.48	10.68	12.58
ST-Mamba	29.89	35.39	40.09	33.88	38.48	45.28	10.75	9.85	10.85
ImPreSTDG	29.21	34.31	38.80	32.98	38.12	41.97	8.12	9.42	10.62

**Table 5:** Prediction performance across different time steps on PeMS04.

Models	PeMS04 (10 min/40 min/60 min)								
	MAE			RMSE			MAPE (%)		
MDGCRN	28.93	35.89	42.89	34.76	41.82	48.82	12.33	13.72	14.33
STHGFormer	30.31	37.40	44.40	35.11	42.11	49.11	13.01	15.01	15.01
FEHGCARN	27.75	34.87	41.87	33.44	40.52	47.52	11.57	13.21	13.07
MMNCCRN	29.75	36.82	43.82	34.97	42.04	49.04	12.74	14.36	14.74
ST-MambaSync	26.11	33.19	40.19	32.17	39.23	46.23	10.51	12.17	12.01
ST-Mamba	28.53	35.63	42.63	34.88	42.01	49.01	11.53	13.73	13.53
ImPreSTDG	25.50	32.54	39.54	31.98	39.04	46.04	8.09	9.39	10.21

**Table 6:** Prediction performance across different time steps on PeMS07.

Models	PeMS07 (30 min/60 min/120 min)								
	MAE			RMSE			MAPE (%)		
MDGCRN	18.25	26.25	34.25	28.47	37.47	47.47	12.72	13.45	13.67
STHGFormer	19.62	27.62	36.62	30.85	39.85	49.85	14.28	15.34	15.28
FEHGCARN	18.87	26.87	34.87	29.13	38.13	48.13	12.21	11.56	11.89
MMNCCRN	17.08	25.08	33.08	27.59	36.59	46.59	13.36	12.19	12.03
ST-MambaSync	16.35	24.35	32.35	26.88	35.88	45.88	11.17	10.63	10.47
ST-Mamba	17.53	25.53	33.53	27.94	36.94	46.94	12.73	11.57	11.82
ImPreSTDG	15.33	23.33	31.33	25.67	34.67	44.67	8.39	7.99	7.83

### Ablation study

**Fig. 12:**
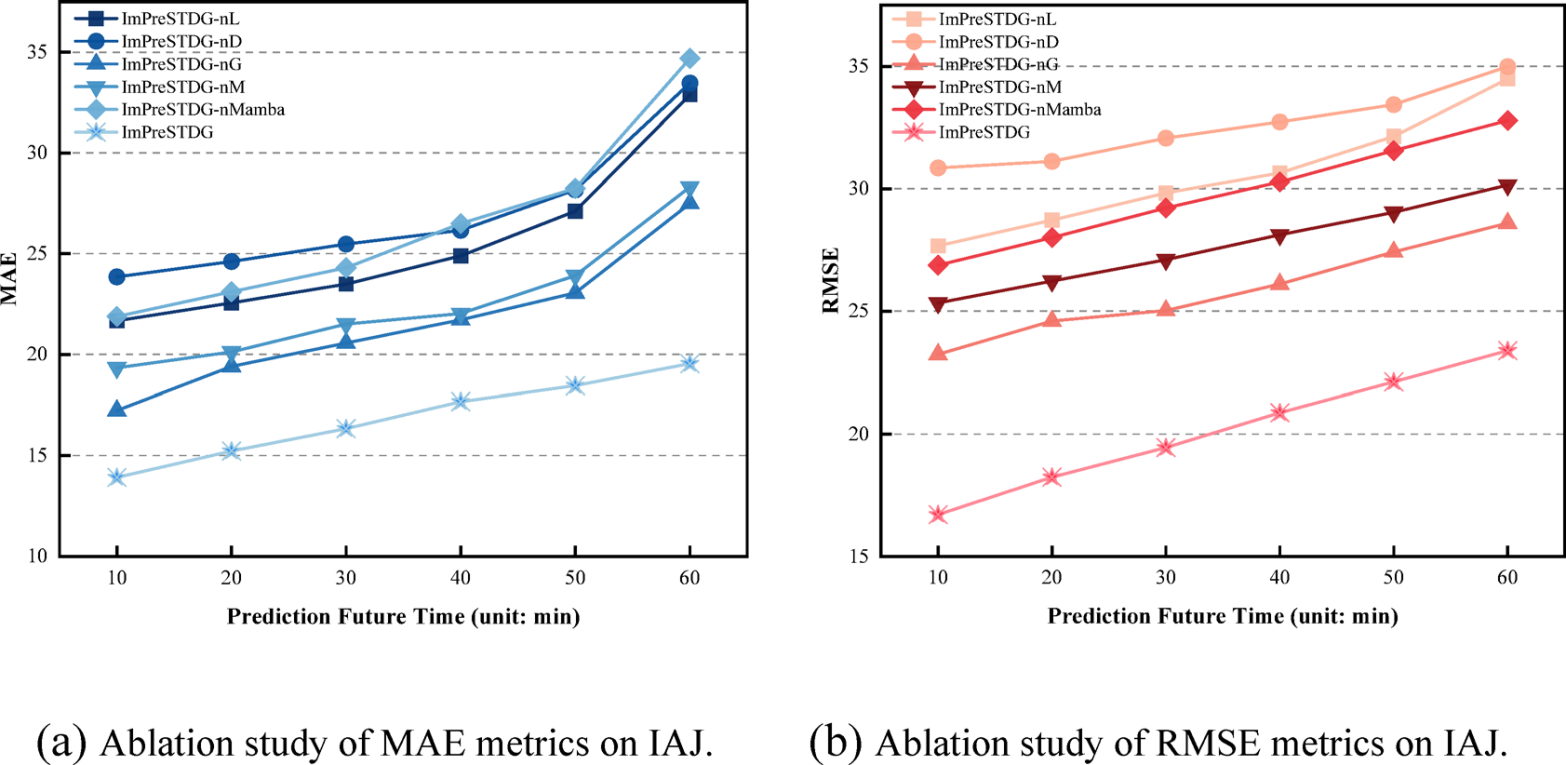
Comparison of performance metrics across ablation study on IAJ.

**Fig. 13:**
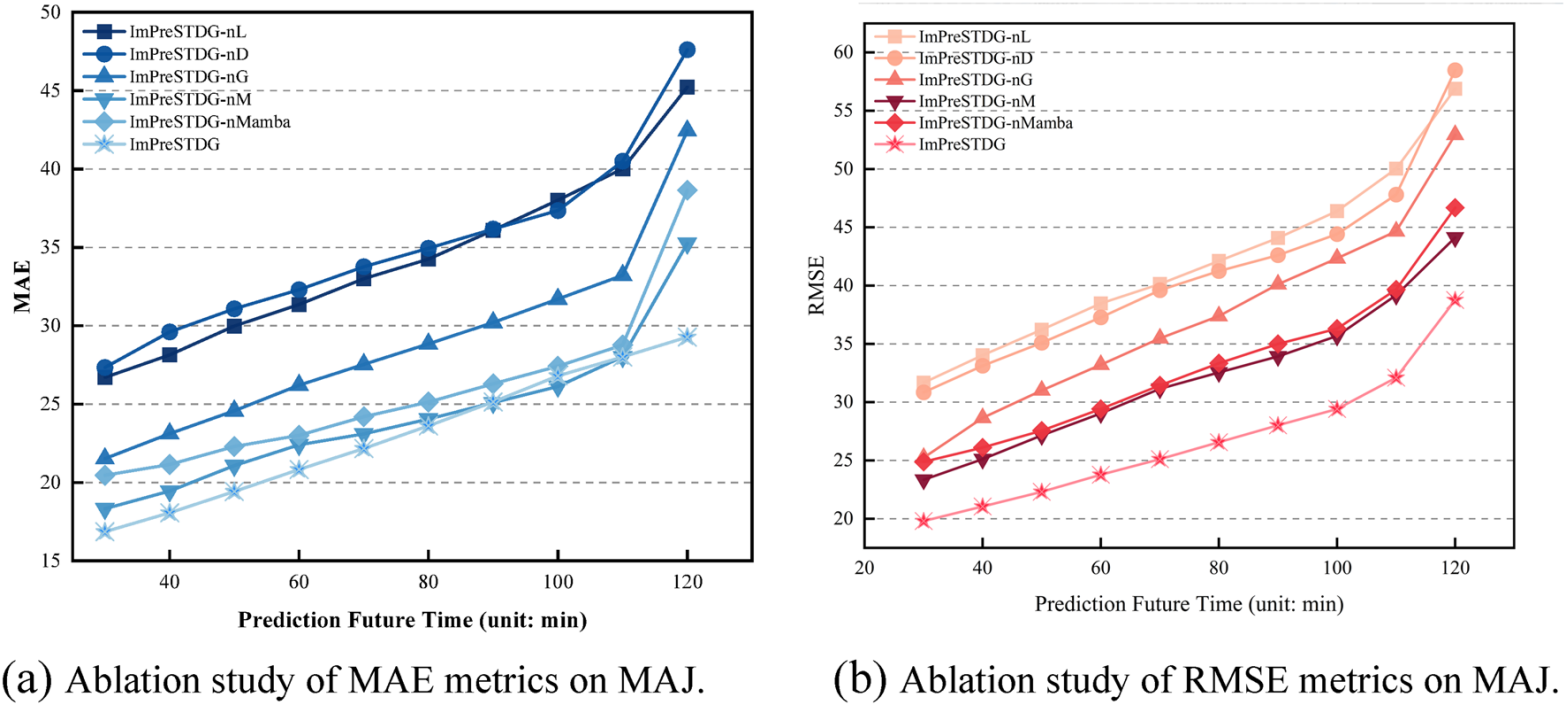
Comparison of performance metrics across ablation study on MAJ.

To further demonstrate that the full model outperforms simplified versions, the performance of model variants with different modules removed is shown in Figs. 12 and 13. Where different variations are listed as followed:

- **ImPreSTDG-nL**: the model with no Long sequence embedding.
- **ImPreSTDG-nD**: the model with no Denoising diffusion probabilistic model.
- **ImPreSTDG-nG**: the model with no GCNs operator.
- **ImPreSTDG-nM**: the model with no Meta fusion module in forecasting header.
- **ImPreSTDG-nMamba**: the model with no Mamba module.

The results show that the full model performs better than any of its abbreviated versions, indicating that each component contributes to improved performance.

### Generalization on different datasets

**Fig. 14:**
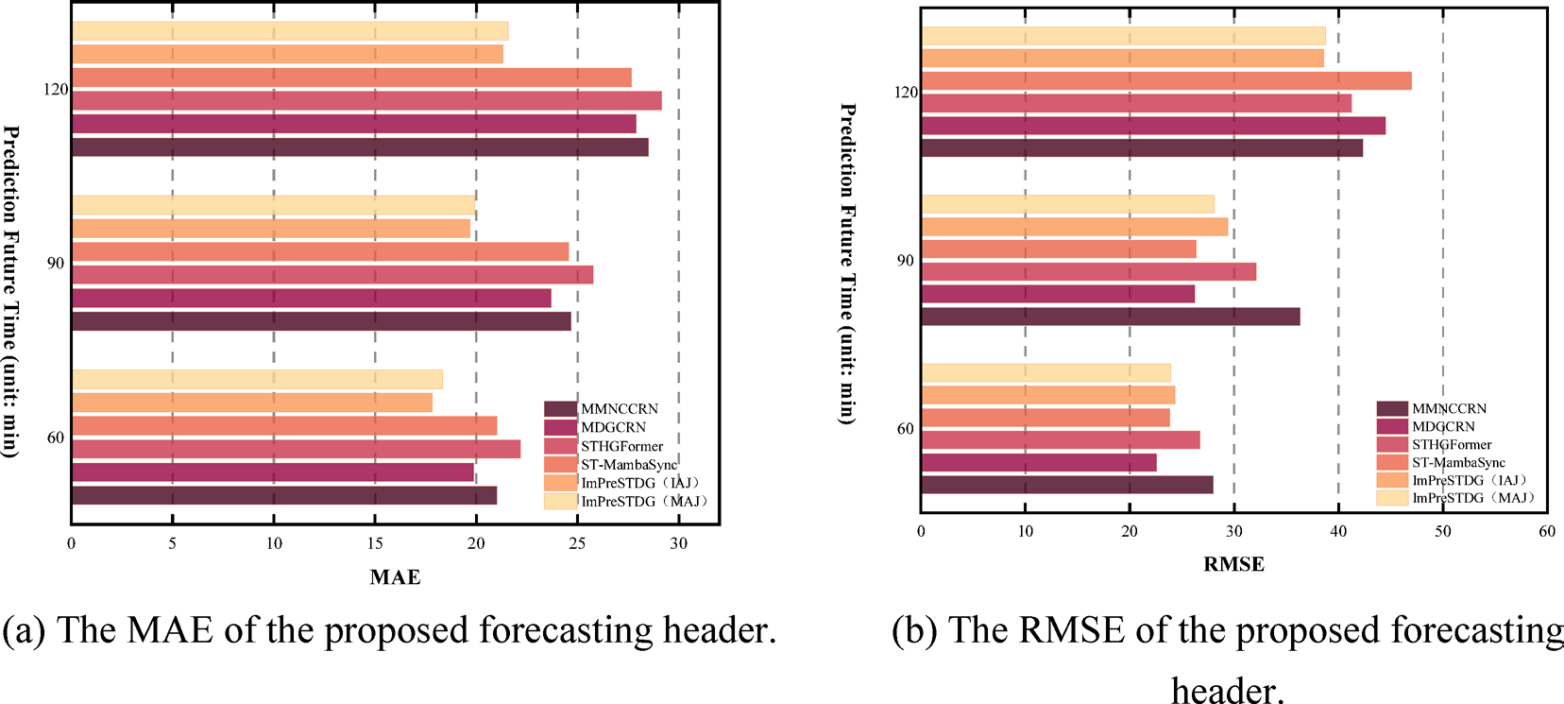
Performance of the proposed forecasting header on different datasets.

**Fig. 15:**
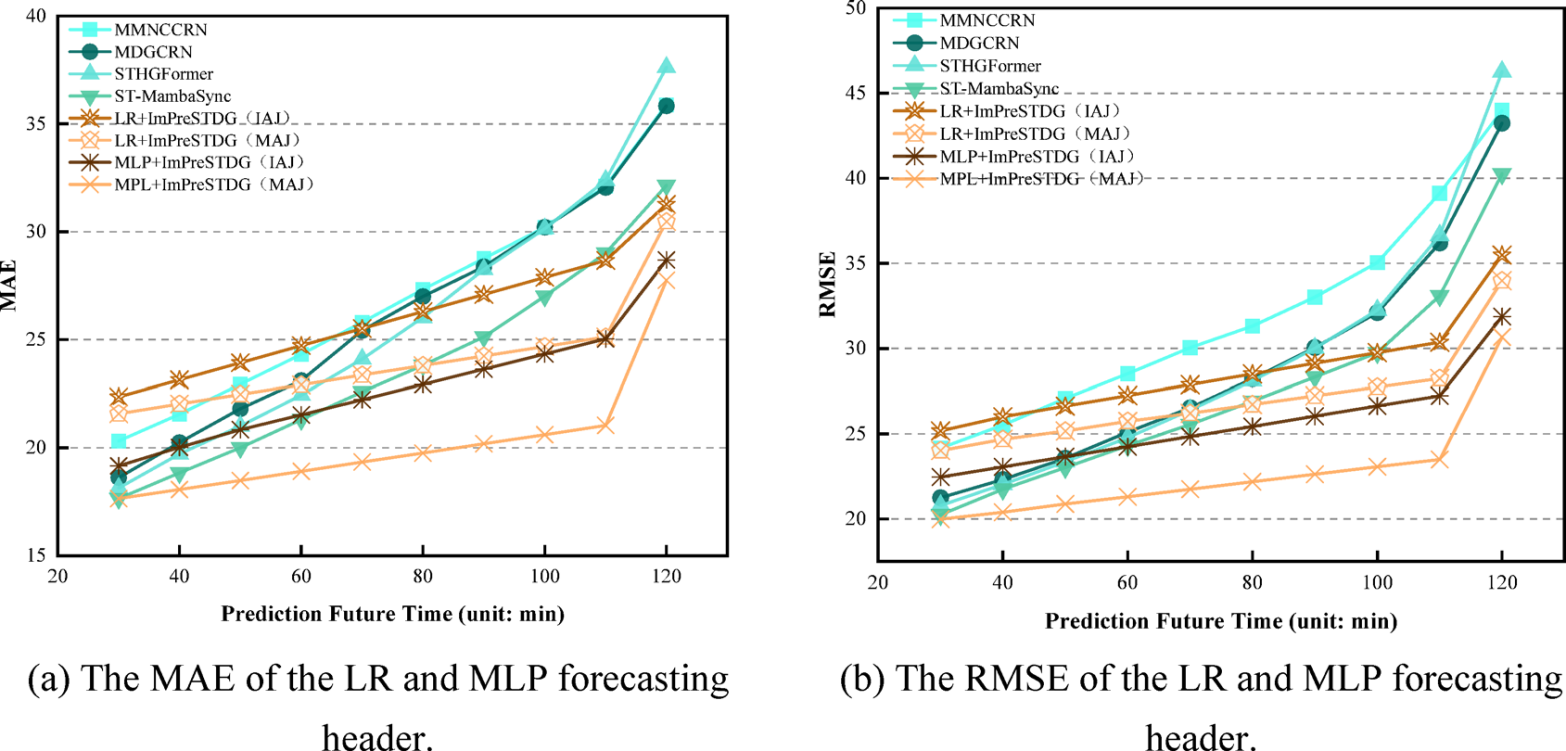
The verification results of backbones pre-trained on IAJ and MAJ.

To explore the generalization ability of the proposed ImPreSTDG method, a natural question arises: can the backbone pre-trained on the other two datasets be reused in MAJ? A case study is conducted to evaluate the accuracy of the pre-trained backbones from IAJ and IMJ on MAJ with different prediction heads. The results are presented in Fig. 14. Fig. 14 illustrates the MAE and RMSE results for the last three time steps. The pre-trained backbones from IAJ and MAJ can be reused in IMJ, outperforming other similar methods. This suggests that pre-trained backbones from high-quality datasets are transferable to other traffic scenarios. On the other hand, the validation results with the backbone pre-trained on IAJ and MAJ are shown in Fig. 15, using linear regression (LR)^38,39^ and multi-layer perceptron (MLP)^40,41^ as prediction heads, tested on IMJ.

### Discussion

While the proposed ImPreSTDG framework achieves competitive forecasting accuracy across multiple benchmark datasets, its broader significance lies in its practical applicability and scalability within real-world urban environments. Architecturally, the model has been purposefully designed to support modular extensibility and computational efficiency, allowing it to be readily adapted to traffic networks of varying sizes and data availability. The incorporation of a SSM—specifically, the Mamba module—ensures that temporal modeling is performed with linear computational complexity relative to sequence length, thereby enabling the handling of long historical traffic records at high temporal resolutions. This contrasts favorably with Transformer-based architectures, which typically suffer from quadratic time and memory costs.

Furthermore, the graph convolutional component operates over sparse adjacency matrices, allowing for efficient parallelization and localized computation—two critical properties for deployment within large-scale sensor networks. These architectural design choices make the model highly suitable for scalable and distributed intelligent transportation systems.

A key consideration for real-world deployment is the adaptability to domain shift, as traffic patterns can vary dramatically across cities due to differences in topology, population density, and environmental conditions. The pretraining mechanism embedded in ImPreSTDG addresses this challenge by learning robust and transferable spatiotemporal priors through denoising-based reconstruction tasks. This unsupervised strategy enables the model to acquire generalizable representations that can be fine-tuned with minimal supervision when applied to new domains. Additionally, the framework supports flexible reconfiguration of key parameters—such as sequence length and input dimensionality—without necessitating full retraining, making it adaptable to heterogeneous sensing infrastructures.

The robustness of the model to noisy or incomplete data is another crucial advantage. In operational deployments, traffic data is frequently affected by missing values, sensor malfunctions, and non-stationarities caused by external disruptions such as inclement weather or construction. The DDPM module mitigates these issues by learning to reconstruct corrupted or masked input sequences, a process that is both label-free and self-supervised, thereby facilitating continuous adaptation as new, unlabeled data becomes available and reducing long-term model maintenance costs.

From a deployment standpoint, the framework supports an offline pretraining–online inference paradigm. The computationally intensive pretraining phase can be executed on cloud infrastructure, while the lightweight forecasting head and SSM modules can be deployed at the edge to enable low-latency, real-time inference. This architecture aligns well with the distributed nature of modern transportation systems, ensuring predictions are made closer to the data source. Moreover, the modular design of the forecasting head allows it to be tailored to specific prediction tasks—such as traffic speed, volume, occupancy, or incident likelihood—thus supporting a broad array of smart mobility applications including signal control optimization, dynamic tolling, and emergency response planning.

Despite its strengths, the current framework is subject to certain limitations. The model presently operates on a static graph structure, which may be insufficient for capturing time-varying changes in road connectivity, congestion patterns, or temporary disruptions. Future research could explore the incorporation of dynamic graph construction mechanisms or adaptive edge weighting schemes. Additionally, while the DDPM component enhances noise robustness, its iterative sampling mechanism remains computationally intensive. Future work may benefit from accelerated sampling techniques or the integration of score-based diffusion alternatives to further reduce inference latency and improve deployment feasibility.

By embedding considerations of efficiency, adaptability, and robustness directly into the model’s architecture, ImPreSTDG moves beyond algorithmic novelty to offer a practical and extensible solution for real-world traffic forecasting. Its dual-path learning structure—combining generative pretraining with efficient state-space inference—positions it as a strong candidate for integration into next-generation intelligent transportation infrastructures, where predictive accuracy, resilience, and domain adaptability must be achieved simultaneously.

## Conclusion

An enhanced pre-training method is introduced in this study to overcome the limitations of traditional graph neural networks in traffic forecasting. The model’s intrinsic robustness is improved without the need for extensive dataset pre-processing, unlike other methods. To fully capture long-term spatiotemporal relationships in traffic data, the proposed model includes a backbone network for long-term data processing and a prediction header for short-term data. Additionally, a pre-training strategy is designed to strengthen the backbone structure and enable the generation of generalized traffic features. Experimental results on three real-world datasets demonstrate that the proposed ImPreSTDG performs well across different scenarios. More importantly, the pre-trained backbone is compatible with various prediction headers, and when trained on high-quality data, it can be deployed on other datasets as a ready-made toolbox, highlighting the method’s importance and universality.

In future work, we will continue to focus on traffic forecasting, particularly the impact of traffic accidents. We plan to develop a “detection sentinel” mechanism to navigate and identify sudden events within the graph structure created from road information, such as changes in road network structure or node relationships due to road maintenance or traffic accidents.

## Data Availability

Data and code will be available upon request. Contact the corresponding author at kangyuyun@lyu.edu.cn.
